# Seasonal and Spatial Variations of Saltmarsh Benthic Foraminiferal Communities from North Norfolk, England

**DOI:** 10.1007/s00248-016-0895-5

**Published:** 2016-11-26

**Authors:** Salha A. Saad, Christopher M. Wade

**Affiliations:** School of Life Sciences, University of Nottingham, University Park, Nottingham, NG7 2RD UK

**Keywords:** Benthic foraminifera, Seasonal trend and spatial distribution, GAM analyses, Saltmarshes

## Abstract

Time series foraminiferal data were obtained from samples collected from three sites at Brancaster Overy Staithe, Burnham Overy Staithe and Thornham on the North Norfolk coast over a 1-year period. At each collection point, six environmental variables—temperature, chlorophyll, sand, mud, pH and salinity—were also measured. The principle aim of this study was to examine the benthic foraminiferal fauna in regard to the temporal variability of foraminiferal abundance, seasonal trend, dominant species, species diversity and the impact of environmental variables on the foraminiferal communities in the top 1 cm of sediment over a 1-year time series. The foraminiferal assemblages at the three sites were dominated by three species: *Haynesina germanica*, *Ammonia* sp. and *Elphidium williamsoni*. Foraminiferal species showed considerable seasonal and temporal fluctuation throughout the year at the three investigated sites. The foraminiferal assemblage at the three low marsh zones showed a maximum abundance in autumn between September and November and a minimum abundance observed between July and August. There were two separate peaks in the abundance of *Ammonia* sp. and *E. williamsoni*, one in spring and another in autumn. In contrast, *H. germanica* showed a single peak in its abundance in autumn. A generalized additive modelling approach was used to explain the variation in the observed foraminiferal abundance and to estimate the significant impact of each of the environmental variables on living foraminiferal assemblages, with taxa abundance as the dependent variable. When included in the model as predictors, most of the environmental variables contributed little in explaining the observed variation in foraminiferal species abundance. However, the hypotheses for differences amongst sites, salinity and pH were significant and explained most of the variability in species relative abundance.

**Electronic supplementary material:**

The online version of this article (doi:10.1007/s00248-016-0895-5) contains supplementary material, which is available to authorized users.

## Introduction

Shallow coastal habitats are considered as dynamic environments characterized by spatial heterogeneity and subject to continuous disturbance. This is in particular the case of the intertidal zone where different environments are developed as a result of exposure to the tide regime [1, 2]. The majority of intertidal environments can be categorized according to plant communities, tidal range and depositional regime into three zones: high marsh, low marsh and tidal flats [3, 4]. Benthic foraminifera are one of the inhabitants of the intertidal environments and have been extensively studied [3, 5]. They vary seasonally and spatially in a way that reflects the fluctuation in biotic and abiotic environmental variables [3, 6–8], and different environmental conditions can lead to different relative abundance and assemblage compositions of foraminiferal species. There is a great deal of interest in information on environmental factors that impact the development of foraminiferal assemblages in the intertidal communities [4, 9–12], with studies mainly centred on the interaction between the biological communities and their physical and chemical environment and the causes of the observed changes [1, 2, 6]. It is commonly believed that changes in specimen abundance are controlled by season which drives cyclic changes of environmental variables [6].

The distribution of benthic foraminiferal species is influenced by a broad range of physical, chemical and biological parameters such as tidal cycle, temperature, salinity, depth, sediment, oxygen, saltmarsh vegetation and food [3, 6–8]. Organic carbon, nitrogen and bacterial activities may also have a minor influence [13]. Comparisons of different intertidal environments have shown that fluctuations in salinity and elevation have the most influential effect on the foraminiferal distribution and zonation pattern [3, 4, 14]. Salinity fluctuation is a result of alternating periods of inundation, desiccation, heavy rain and river input [2, 3]. Temperature, food availability and grain size of the sediment are also often considered to have a great influence on the distribution of foraminiferal assemblages [8, 14]. However, it must be remembered that the significance of individual environmental factors varies seasonally and spatially and that different factors might be more significant at different times [8]. On the other hand, it is often assumed that the abundance and distribution of species of benthic foraminifera are largely determined by biological interactions such as predation and competition [2], indicating that the reproduction rate of one species is probably inhibited by competing species [15].

To reveal the seasonal variation in abundance and diversity and the environmental conditions associated with it, a time series study of the living foraminiferal assemblage was undertaken over a period of 1 year, with samples collected on a monthly basis from three low marsh sites, Brancaster Overy Staithe, Burnham Overy Staithe and Thornham, on the North Norfolk coast. North Norfolk is characterized by a low upland separated from sand and shingle beaches by extensive saltmarshes and intertidal flats [16]. The intertidal zone of North Norfolk has been described as the finest area of coastal marsh in Great Britain [17]. It covers a broad range of environments including tidal flats and low, middle and high marshes. The three selected sites, Brancaster Overy Staithe, Burnham Overy Staithe and Thornham, have been known for their richness in foraminiferal assemblage and provide excellent locations for following the seasonal changes in foraminiferal biodiversity. Although considerable research has been directed towards the study of intertidal communities and the physical and chemical processes that support them in a number of intertidal environments around the coastline of Great Britain [4, 6, 18–22], this study provides the most extensive survey to date of living foraminiferal taxa from the low intertidal zone on the North Norfolk coast when the monthly sampling of specimens over a 1-year period is taken into account. It is known that the foraminiferal assemblage has stronger associations with certain habitat types on the intertidal zone, which makes them a potential indicator of wider changes in biodiversity within those habitats and a good indicator of ecosystem health. This type of study will ultimately contribute to our understanding of the variability and cyclicity in the abundance and the rate of accumulation of foraminiferal tests in the sediment [23, 24]. Substantial documentation of the dominant and main species and the occurrence of rare taxa that are present on every sampling occasion will also be obtained.

We have also assessed the variation in foraminiferal abundance and species composition through the construction of ecological models. These include hypotheses to test the significance of differences between sites and seasons as well as hypotheses to test whether species composition and abundance are determined by the measured environmental variables. A generalized additive model was run using environmental data as predictors and foraminiferal abundance data as a response variable. It is a nonparametric regression analysis that is often used to predict nonlinear response of abundance to known environmental settings over a broad geographic area in order to infer the likelihood that a certain species would inhabit a particular environment. The output of these models can then be used to infer the possible environmental drivers of the observed changes and eventually will help in developing the appropriate regional environmental conservation schemes. The serious biases in the analysis of seasonal trends due to the spatial variation of foraminiferal species distribution have been accommodated through considering replicate samples from within each low marsh site to account for the imperfect detectability. It is often suggested that differences in monthly records of abundance may be caused by the patchiness in the distribution patterns which is often seen on 10-cm and 1-m scales [25]. Thus, one of the aims was to assess the significance of spatial variability in foraminiferal assemblage in our seasonal estimate of abundance via incorporation of time series data in stations that are as close as 1 m and are subjected to the same overall environmental conditions.

## Methods

### Study Area

The North Norfolk coast is an extensive site that extends over 50 km in length and includes coastal features such as Scolt Head Island, a large coastal island, and Blakeney Point, a large shingle spit. The region is characterized by wide expanses of fine sand flats, barrier islands, sand and shingle beaches and spits backed by extensive fine-grained, vegetated saltmarshes and large areas of tidal flats and dunes. The North Norfolk coast has a meso- to macrotidal range of approximately 6.4 m at Spring tides and 3.2 m at neap tides. Most of the saltmarshes lie behind coastal barriers of sand (in Brancaster and Titchwell), shingle (Blakeney Point) or mixed sand and shingle (Scolt Head Island). These saltmarshes have vegetation cover of glasswort (*Salicornia* spp.), cordgrass (*Spartina anglica*), and sea aster (*Aster tripolium*), sea purslane (*Atriplex portulacordes*), sea lavender (*Limonium vulgare*) and sea meadow grass (*Puccinellia maritime*). The three studied sites, Brancaster Overy Staithe, Burnham Overy Staithe and Thornham, are about 11.7 km apart and cover a broad range of intertidal environments. Brancaster Overy Staithe (latitude 52°58′05.83″ N, longitude 0°40′03.52″ E) is about 2.14 km downstream of the western end of Scolt Head Island whilst Burnham Overy Staithe (latitude 52°57′55.56″ N, longitude 0°44′48.59″ E) is at about 1.67 km downstream of the eastern end of the island. Scolt Head Island is a major barrier island with a sand and shingle beach along the north coast and recurved spits to the south which encloses saltmarshes. The saltmarsh habitats are around 7.7 km wide and deeply dissected by multiple creeks and tidal channels. They can be divided into high, middle and low marsh and tidal flats on the basis of vascular flora. Brancaster Overy Staithe and Burnham Overy Staithe collection sites are located on muddy low intertidal zone (low marsh) where two plant species, *Festuca ovina* and *Salicornia europaea*, dominate and are adjacent to the harbours. Grain size analysis showed that the sediment from both localities was muddy sand sediment composed of 31–34 % mud and 66–69 % sand. Salinity varies significantly between tides (5–30 ‰) as a result of freshwater discharge from the River Burn in Burnham Overy Staithe and from freshwater springs in Brancaster Overy Staithe. Thornham is located on Brancaster Bay at a latitude of 52°57′59.37″ N and longitude of 0°34′20.16″ E and about 1.5 km inland. A series of different saltmarsh environments, upper saltmarsh, tidal channel, saltmarsh, dunes and beach, are present on Brancaster Bay. The sampling point at Thornham was located near the head of a creek on a mud bank. The sediment consisted of a thick and soft layer of mud with 68 % mud and 32 % sand, with this site classified as having sandy mud sediment.

### Foraminiferal Sampling Procedure

**Fig. 1:**
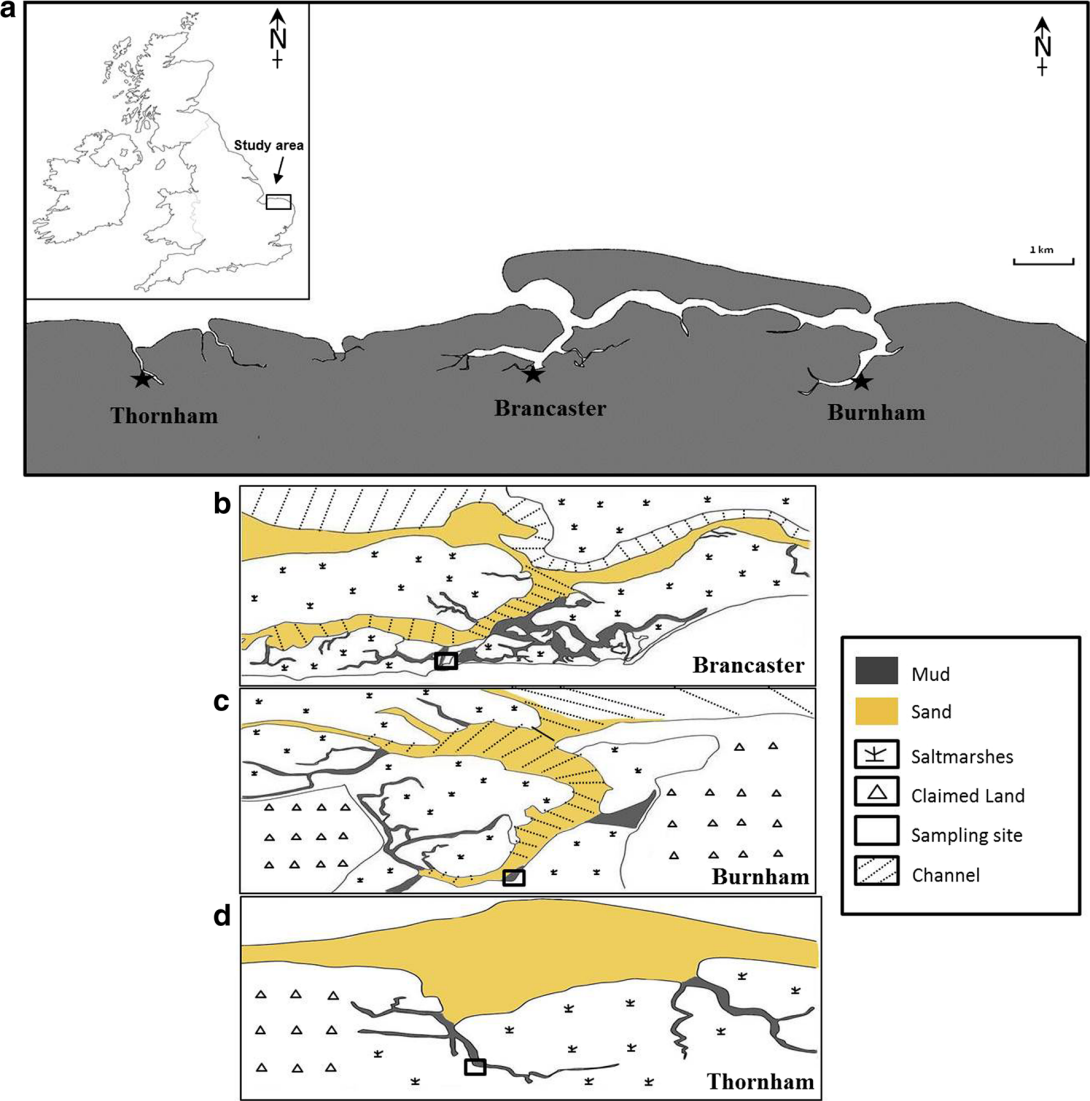
Study area map with the three sampling sites. **a** North Norfolk coast. **b** Brancaster Overy Staithe. **c** Burnham Overy Staithe. **d** Thornham

Sampling was carried out on a monthly basis for a period of 1 year from January 2012 to January 2013. At each of the three sites—Brancaster Overy Staithe, Burnham Overy Staithe and Thornham (Fig. 1)—three replicate samples were collected for foraminiferal abundance study, three for chlorophyll measurements, three for sediment size analysis and three for salinity and pH measurements. Air and mud temperatures were recorded at the time of sample collection. The three replicate samples were 1.5 m apart and with the same elevation and the same length of subaerial exposure. The total living benthic foraminiferal assemblage was examined at each station. All sediment samples for foraminiferal analysis were collected around low tide as follows. The uppermost layer (1 cm) of sediment was collected by pressing a 53-mm plastic Petri dish with volume of 22 cm^3^ and 1-cm depth into the mud. The Petri dish was then lifted out of the sediment by sliding a metal plate underneath. Samples were then wrapped in plastic bags and brought immediately to the lab. In the laboratory, the entire sample was removed from the Petri dishes and preserved in a Duran bottle containing 100 % ethanol for 24 h. Each sample was then washed through a 53-μm sieve with tap water and then stained with 20 ml of 1 % Rose Bengal in the Duran bottle overnight in order to differentiate between living and dead foraminifera. The sediment was washed again to remove the surplus stain and dried at 60 °C overnight. A sieve size of 53 μm was used to ensure that small opportunistic taxa below the 125-μm size fraction were not lost. We note that the use of Rose Bengal staining may lead to a slight overestimation of the living assemblages [26] as Rose Bengal is protein-specific and may stain proteins still in the shell after death [27]. Dried sediment samples were then brushed through a 1-mm sieve to disaggregate the organic contents. The sediment sample from each replicate was examined under a binocular microscope in its entirety and every individual stained benthic foraminiferal specimen that retained a pink colour was identified based on morphology and counted. In total, 79,457 individuals were counted in this study, and an average of 679 individuals were found in each replicate.

### Environmental Variables

#### Determination of Chlorophyll

The value of chlorophyll of the top 0.5 cm of the sediment has always been considered as a good indicator of the availability of food for foraminifera [6]. The chlorophyll content of the top 1 cm of sediment at the three study sites was measured according to Parsons et al. [28] and as follows: a volume of 22 cm^3^ was sampled from the top 1 cm of the sediment using a 53-mm Petri dish following the same procedure as in foraminiferal sampling. Three replicates a few centimetres away from the foraminifera sampling points were taken per site, stored in a container away from the light and brought immediately to the lab. The samples were washed with seawater into a 50-ml centrifuge tube, centrifuged at 3000 rpm for 10 min and the water decanted. Twenty-five millilitres of 90 % of acetone was added to the sediment, shaken thoroughly and allowed to stand in the dark in a fridge at 4 °C for 24 h. The content of each tube was centrifuged at room temperature for 10 min at 3000 rpm. Four millilitres of the supernatant was decanted into a spectrometer cuvette (1-cm path length) and measurements were taken at a wavelength of 665 (chlorophyll a). The spectrophotometer was zeroed using 90 % acetone. A measurement at 750 nm as a turbidity blank was also taken and then subtracted from the reading of other wavelengths for correction. The active chlorophyll a in the sediment samples was calculated after correction for the phaeopigment products using the following formula:

$$ \mathrm{Chlorophyll}\kern.2em a\kern.2em \left( mg/{m}^3\right)=\frac{26.7\ \left({665}^{\circ }-{665}^{\mathrm{a}}\right)\times v}{V\times l} $$

where 665° is the extinction at 665 nm before acidification, 665^a^ is the extinction at 665 nm after acidification, *V* is the volume of sediment sampled (22 cm^3^), *v* is the volume of acetone in millilitres (25 ml) and *l* is the path length (1 cm) of the cuvette.

#### Measurement of Sediment pH and Salinity

Sediment pH and salinity were measured from samples of volume of 63.6 cm^3^ that were collected from each site with three replicates each at the time of benthic foraminifera sample collection following the methods of Taworn and Boyd [29, 30], in which the dried sediment is resuspended in distilled water and the resulting pH and salinity are measured in the supernatant. These replicates were taken about 5 cm apart from the foraminifera sampling points. Sediment samples were brought immediately to the lab, dried at 60 °C in the oven and pulverized to pass a 2-mm sieve. A mixture of 1:2.5 soil to water was then made using distilled water and stirred with a glass rod for 30 min. pH values were measured by inserting pH electrodes into the mixture whilst stirring and a reading was taken. The mixture was then stirred at regular intervals for 1 h and allowed to settle down for 20 min. The supernatant was filtered through a dry Whatman no. 42 filter paper into a dry beaker and a salinity reading was taken by inserting a salinity meter into the filtered supernatant.

#### Sediment Size Analysis

The percentages of different sediment size particles were measured from 0.5 g air-dried sediment samples. The dried sediment samples were initially sieved to <2 mm in size and 0.5 g was transferred into a 50-ml centrifuge tube. Soil organic matter was chemically removed from the soil using 25 ml of hydrogen peroxide overnight. To ensure all organic matter had been removed from the soil sample, the centrifuge tube was placed in a 60 °C water bath for 1–1.5 h, with the temperature raised to 90 °C for an additional 1–1.5 h. Samples were topped up with 25 ml of deionised water prior to centrifuging at 3500 rpm for 4 min. The remaining solution was decanted off, with an additional 35 ml of deionised water added to the sample prior to centrifuging at 3500 rpm for 4 min once again. The remaining solution was decanted and 25 ml of calgon (35 g of sodium hexametaphosphate, 7 g sodium carbonate in 1 l of deionised water) added before continually shaking the sample prior to analysis. Prior to analysis, the sample was placed in an ultrasonic bath for 30 min to keep all soil particles dislodged. The samples were then analysed and the percentages of different sediment size particles were measured using a Beckman Coulter LS 200 analyser.

### Statistical Analysis

The relationship between the relative abundance of each of the common foraminiferal species at the three examined sites (Brancaster Overy Staithe, Burnham Overy Staithe and Thornham) and six measured environmental variables (predictors or explanatory covariates) was modelled statistically using a generalized additive modelling approach. A generalized additive model (GAM) is a nonparametric regression analysis that relaxes the normality assumptions and allows the nonparametric modelling of predictors in addition to the linear and polynomial terms for other predictors through the use of link functions [31]. The GAM approach is a major extension of the familiar general linear model [32] and the recent generalized linear model [33]. All models were fitted using the mgcv package in the R environment (version 3.1.1) following the equation

$$ \begin{array}{l}\mathrm{g}\mathrm{a}\mathrm{m}\;\left(\mathrm{speciesstandingcrop} \sim \mathrm{s}\left(\mathrm{dayinyear}\right)+\mathrm{Site}2+\mathrm{s}\left(\mathrm{P}\mathrm{H}\right) + \mathrm{s}\left(\mathrm{Temperature}\right) + \mathrm{s}\left(\mathrm{Mud}\right) + \right.\hfill \\ {}\left.\mathrm{s}\left(\mathrm{Sand}\right) + \mathrm{s}\left(\mathrm{Chlorophyll}\right) + \mathrm{s}\left(\mathrm{Salinity}\right),\ \mathrm{random}=\mathrm{list}\left(\mathrm{Replicate}2=1\right),\;\mathrm{data}=\mathrm{Data}\right)\hfill \end{array} $$

where s stands for thin-plate regression spline fitting method for a given environmental variable. The three replicate samples within each site were considered as random effects. GAM fit and variable selection were basically evaluated using either the approaches that minimize the Akaike information criterion (AIC) [34] or the total explained deviance component as measured with the *χ* ^2^ statistic. Adding or removing each of the environmental variables to or from the fitted model was further assessed using an analysis of deviance, ANOVA. Once the foraminiferal community response is derived by the modelling regression above, its potential distribution and their habitat within the studied area can be predicted. Additionally, three diversity indices including Fisher’s alpha, the Shannon–Wiener index, *H*(*S*), and evenness (E_H_) were measured from the samples collected from the three sites in order to characterize the community and to determine how equally abundant those species are in the foraminifera assemblages. Fisher’s alpha is a diversity index that is defined implicitly by the formula *S* = a*ln(1 + *n*/*a*), where *S* is the number of taxa, *n* is the number of individuals and *a* is the Fisher’s alpha. The Shannon–Wiener index, *H*(*S*), is another diversity index that takes into account the number of individuals as well as the number of taxa. It varies from 0 for communities with only a single taxon to high values for communities with many taxa, each with few individuals, *H* = sum((*n*_*i*_/*n*)ln(*n*_*i*_/*n*)), where *n*_*i*_ is the number of individuals in the *i*th taxon. Evenness (*E*_*H*_) can be calculated by dividing *H*(*S*) (Shannon–Wiener index) by ln*S* (where *S* is the total number of species in the community), *E*_*H*_ = *H*/ln*S*. Evenness assumes a value between 0 and 1, with 1 being complete evenness. Estimation of homogeneity and patchiness in the distribution of foraminiferal species was measured using the index of affinity, I. *A*. _*kj*_ = ∑_*i* = 1_^*n*^ min(*X*_*ki*  ,_  *X*_*ji*_ ), where *i* is the *i*th species, *k* and *j* are samples and *n* is the total number of species. The affinity index calculates the similarity between replicates directly from the percentage data, where the lowest value for each species is summed over all species in the three samples.

## Results

### Species Composition

**Fig. 2:**
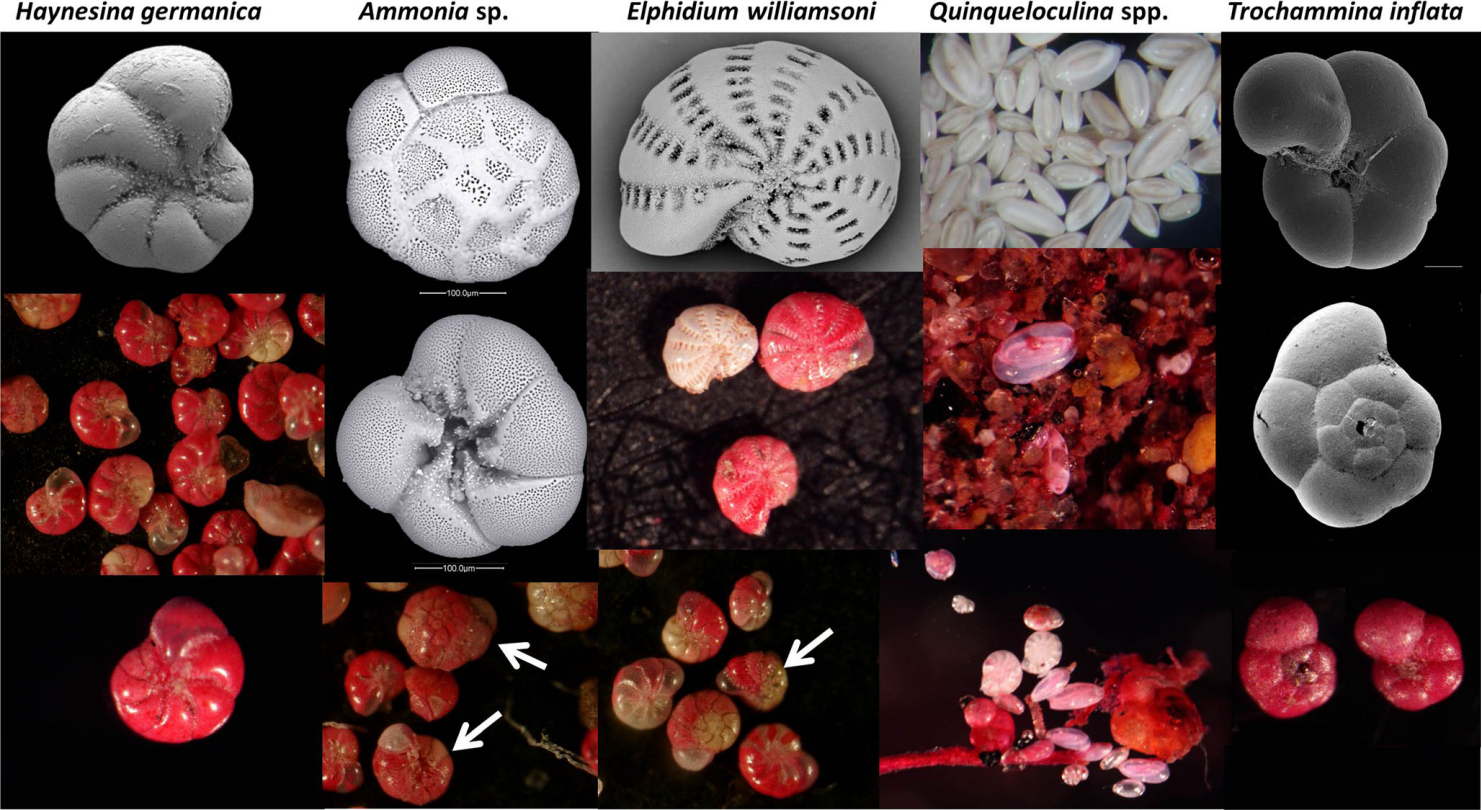
Microscopic images of the five foraminiferal species sampled from the coastline of North Norfolk. *Arrows* point to identified species in pictures where more than one species is shown

**Fig. 3:**
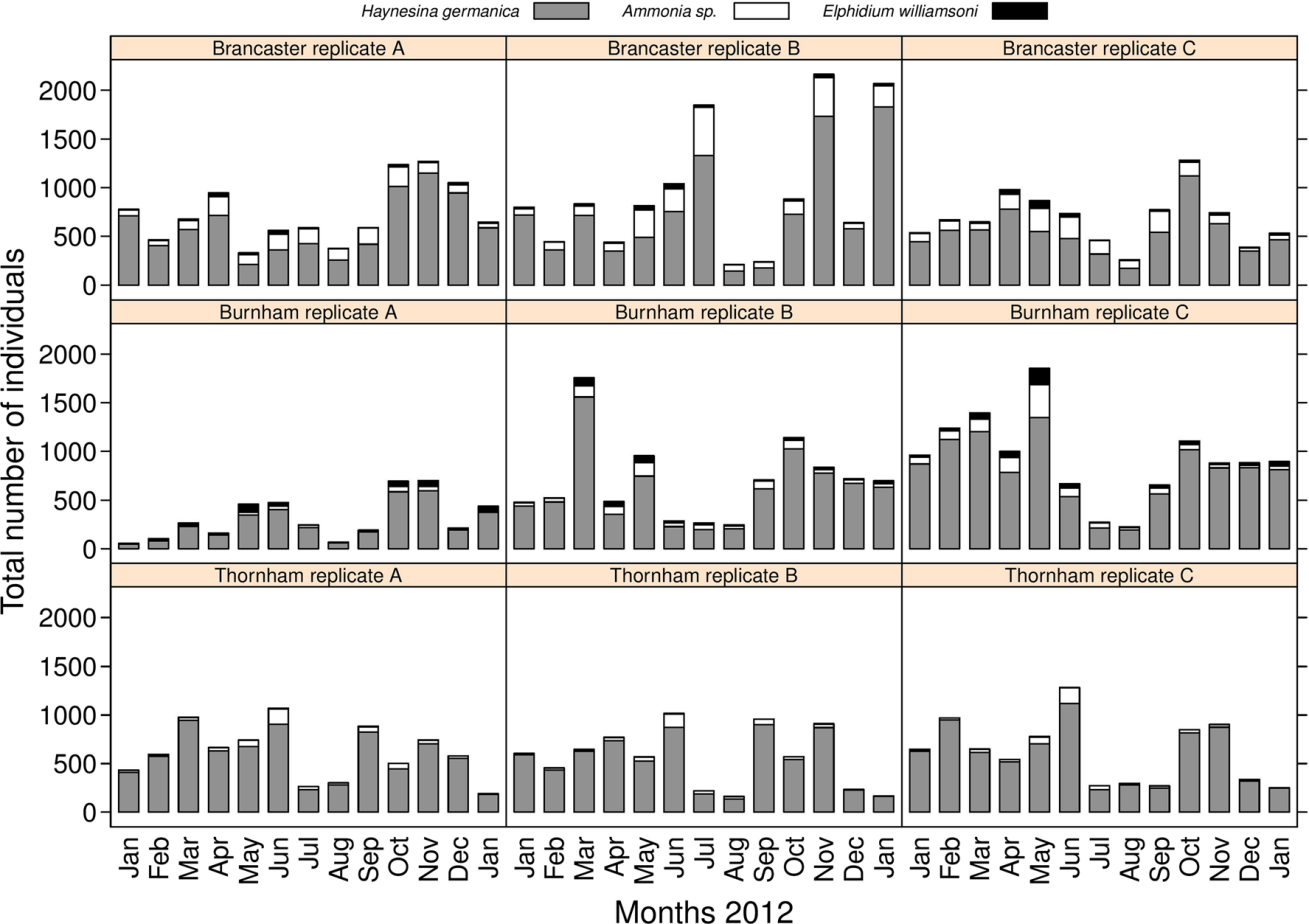
Total abundance and foraminiferal species composition at the three sites and in each replicate

**Fig. 4:**
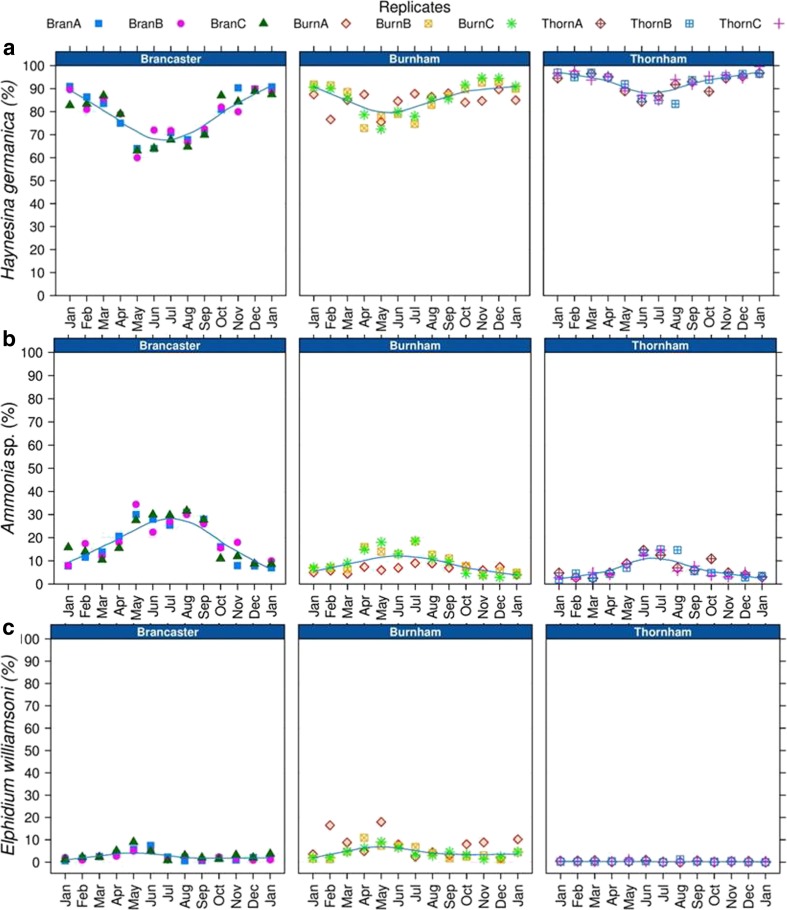
Relative abundance of the three main benthic foraminiferal species at the three investigated sites. **a** *Haynesina germanica*. **b** *Ammonia* sp. **c** *Elphidium williamsoni*

The three main species of benthic foraminifera that occur in the majority of the collected samples from the three sites are *Haynesina germanica* (Banner & Culver, 1978), *Ammonia* sp. (Brunnich, 1772) and *Elphidium williamsoni* (Haynes, 1973) (Figs. 2, 3 and 4 and Tables S2 and S3). An additional calcareous species, *Quinqueloculina* sp. (Linnaeus, 1758), and an agglutinated species, *Trochammina inflata* (Montagu, 1808), are occasionally present as rare specimens (Fig. 2). *H. germanica* dominates the fauna in Brancaster Overy Staithe, Burnham Overy Staithe and Thornham, with relative abundance of 85.6 %, and makes a significant contribution to the total living foraminiferal assemblages throughout the year (Figs. 3 and 4a). *H. germanica* is followed in abundance by *Ammonia* sp. and *E. williamsoni* (relative abundances of 11.4 and 2.8 % of the total assemblage throughout the sampling period, respectively; Figs. 3 and 4b, c). *Ammonia* sp. tends to be more common in Brancaster Overy Staithe than the other two sites (Burnham Overy Staithe and Thornham) and makes up 17.5 % of the total foraminiferal fauna, whilst the highest relative abundance of *E. williamsoni* (5 %) amongst the three sites was reported in Burnham Overy Staithe (Figs. 3 and 4b, c).

### Comparison of Replicate Samples

In this study, foraminiferal data were analysed from samples collected monthly from three sites on the North Norfolk coast, with three replicates at each, over a period of 1 year. In order to test for homogeneity or patchiness in species composition in the data, the three replicate samples were compared using the index of affinity [35]. The level of similarity was expressed as a percentage for each of the three replicate samples at each sampling point (Fig. S1). The affinity index between the replicate samples ranged from 89 to 97 % in Brancaster, from 83 to 97 % in Burnham and from 89 to 99 % in Thornham. It has been claimed that a value of >80 % is indicative of a high degree of similarity [23] (Fig. S1 and Table S1).

### Seasonal Trend of Abundance

#### Seasonal Trend of All Foraminiferal Species

**Fig. 5:**
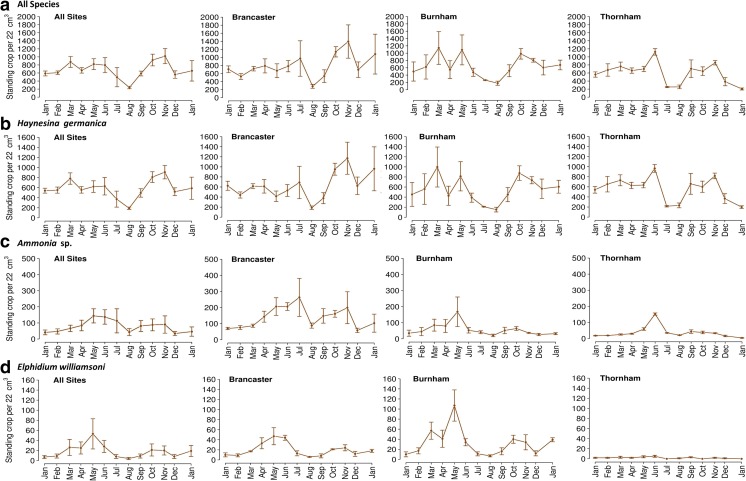
Seasonal trends of the average abundance of foraminifera (all species) and of *Haynesina germanica*, *Ammonia* sp. and *Elphidium williamsoni* individually at each of the three sampled sites. **a** All species. **b** *Haynesina germanica*. **c** *Ammonia* sp. **d** *Elphidium williamsoni. Bars* are ±1 standard deviation from the mean. Abundance reflects foraminiferal abundance per 22 cm^3^

**Fig. 6:**
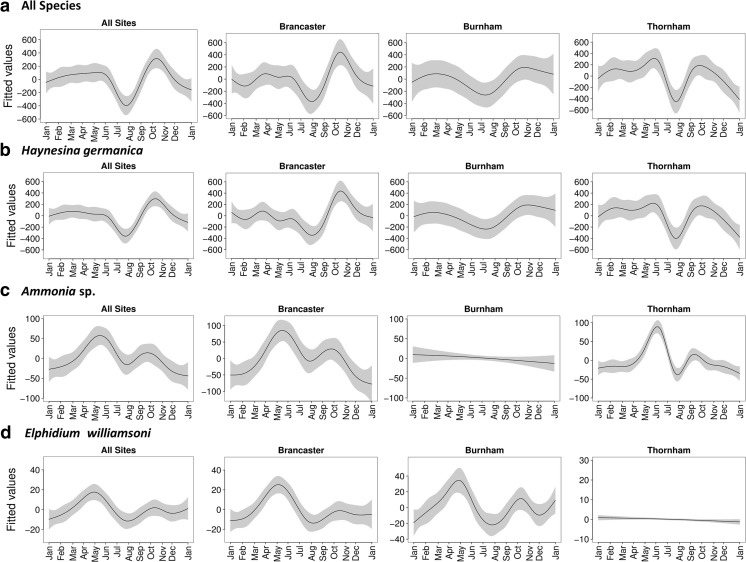
Seasonal trends of the predicted abundance of foraminifera (all species) and of *Haynesina germanica*, *Ammonia* sp. and *Elphidium williamsoni* individually at each of the three sampled sites and the general trend from the whole North Norfolk coast from the GAM analysis. The *line* is the predicted value of foraminifera abundance from the model and the *grey area* represents 95 % confidence intervals. **a** All species. **b** *Haynesina germanica*. **c** *Ammonia* sp. **d** *Elphidium williamsoni*. The *Y*-axis is the fitted values of species abundance with a mean value of zero

Seasonal variation in foraminiferal abundance from January 2012 to January 2013 is shown in Figs. 5 and 6. Although the three sites showed a consistent annual pattern in the mean abundance (the total number of individuals per unit of area of sediment), total foraminiferal abundance across the sampled period was relatively higher at Brancaster Overy Staithe, followed by Burnham Overy Staithe and Thornham (30,872, 25,234 and 23,351 individuals, respectively). The foraminiferal seasonal trend was less variable between the three replicates within each site. The gradual increase in the total number of individuals was observed early in the spring, from March through April and May, reaching maximum values in June (Figs. 5a and 6a). There was then a noticeable reduction between July and August before foraminiferal numbers increase dramatically to their highest values in autumn, between October and November (Figs. 5a and 6a). Foraminiferal abundance then dropped in December 2012 and January 2013 to a number similar to that in January 2012, indicating that the annual cycle might repeat itself.

#### Seasonal Trend in Individual Species

All three species showed a pattern of high relative abundance of individuals in autumn, between October and November, and in spring, between March and May. The highest relative abundance amongst the encountered foraminiferal species in all three sites was for *H. germanica* (Figs. 3 and 4). As this species contributes significantly to the total fauna, its seasonal trend did not vary considerably from the general trend of all foraminiferal fauna throughout the year (Figs. 5b and 6b). *H. germanica* has a steady abundance, with minor changes at the three sites from January until June, when there was a large drop in the number of individuals between July and August (Figs. 5b and 6b). Following this, the highest abundance has then been recorded between October and November. The abundance of *H. germanica* dropped afterwards to roughly the same level at the beginning of the year (Figs. 5b and 6b). This trend was more or less uniform across the three replicates within each site. Despite the noticeable reduction between July and August for all foraminiferal species, they differed in the precise times of peak abundance. For example, the largest peak in abundance of *Ammonia* sp. was more obviously seen between April and June at Brancaster Overy Staithe and Thornham. At Burnham, the peak for *Ammonia* sp. was poorly developed (Figs. 5c and 6c). *Ammonia* sp. abundance was very low during the winter at all sites, but it increased generally in the late winter and early spring. The relative abundance then reached its maximum in May (Figs. 5c and 6c). Thereafter, there was a major drop in abundance in late summer, around July to August, before another minor peak observed in autumn, between September and October (Figs. 5c and 6c). Though following a similar seasonal pattern, the relative abundance data showed that there is a partial substitution of *H. germanica* by *Ammonia* sp. between May and September. At a consistent lower abundance than the other two species, *E. williamsoni* exhibited roughly the same annual cycle as *Ammonia* sp., with two peaks of abundance between March and June and between October and November, with a large reduction period in between (Figs. 5d and 6d). Again, the March to June peak was the highest throughout the studied period (Figs. 5d and 6d). At Thornham, however, the difference in the relative abundance of *E. williamsoni* and its seasonal trends were less pronounced throughout the year (Figs. 5d and 6d).

### Species Diversity

**Fig. 7:**
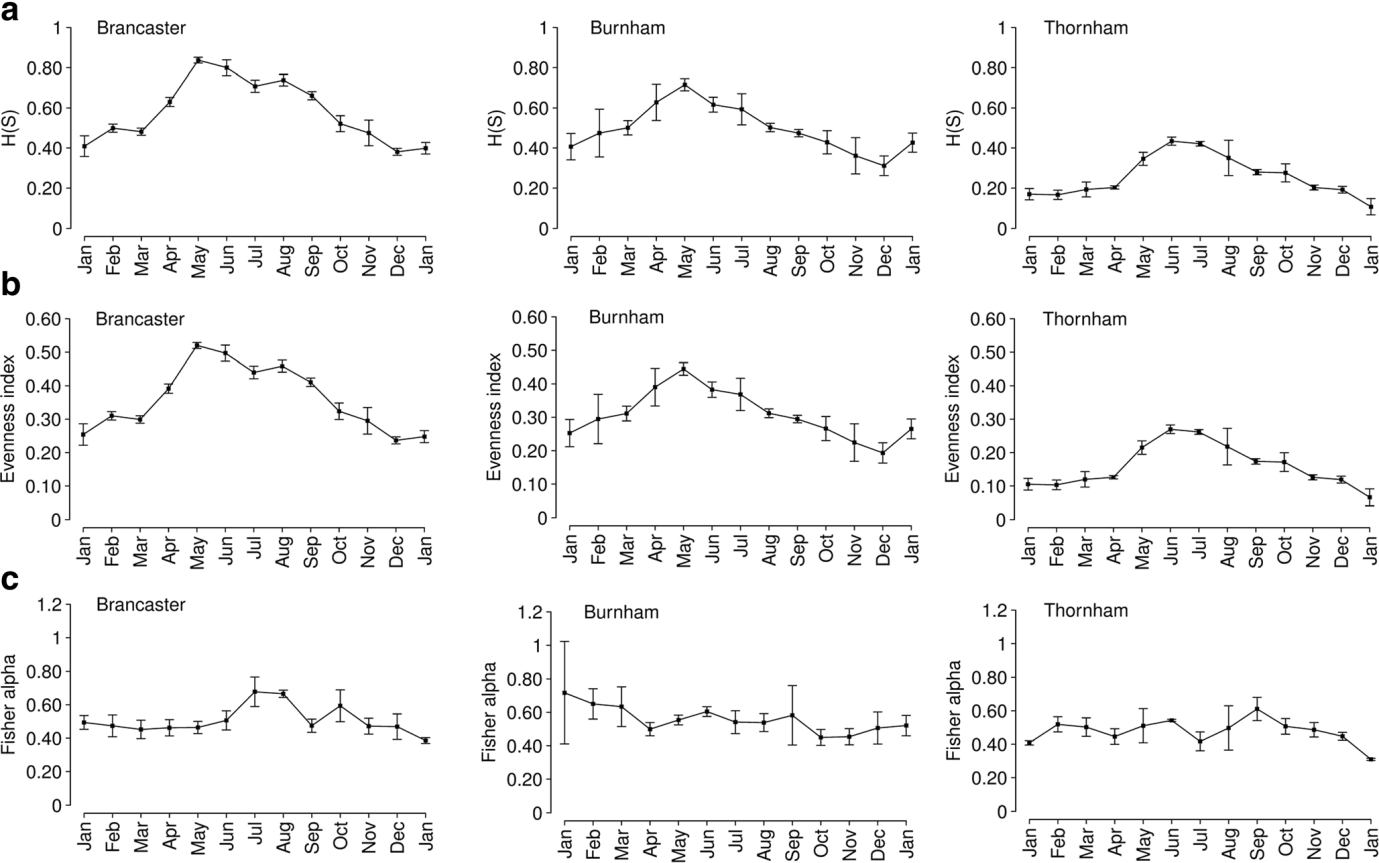
Diversity indices for the three sites, Brancaster Overy Staithe, Burnham Overy Staithe and Thornham. The variability of the diversity indices between replicate samples is illustrated as the average of the three replicates collected at each month for the three sites. **a** Shannon–Wiener index, *H*(*S*). **b** Evenness index (*E*_H_). **c** Fisher’s alpha

Species diversity using three indices, the Shannon–Wiener index, *H*(*S*), Fisher’s alpha and evenness (*E*_*H*_), was measured for the three sites (Fig. 7). The Shannon–Wiener index, *H*(*S*), values at Brancaster Overy Staithe and Burnham Overy Staithe are slightly higher than that at Thornham, suggesting a more diverse foraminiferal assemblage. It ranged from 0.32 to 0.86 (mean = 0.57) at Brancaster Overy Staithe, from 0.23 to 0.77 (mean = 0.49) at Burnham Overy Staithe and from 0.02 to 0.51 (mean = 0.25) at Thornham (Fig. 7a). There was a clear cyclic pattern for the *H*(*S*) values at the three sites, with a gradual increase starting from April through September at Brancaster Overy Staithe, from April through July at Burnham Overy Staithe and from May through August at Thornham. The *H*(*S*) values were generally low before April and after September. Values for evenness (*E*_*H*_) showed identical seasonal variation to *H*(*S*) values, with a gradual increase in late spring and summer months at the three sites. Brancaster Overy Staithe and Burnham Overy Staithe have higher *E*_*H*_ values than Thornham and ranged from 0.20 to 0.53 (mean = 0.36) at Brancaster Overy Staithe, from 0.14 to 0.47 (mean = 0.30) at Burnham Overy Staithe and from 0.01 to 0.32 (mean = 0.15) at Thornham (Fig. 7b). For Fisher’s alpha (the number of species), although there was no obvious pattern over time at the three sites, some samples showed evidence of an increase in spring and autumn. The overall total number of species ranged from 3 to 5 at the three sites. Fisher’s alpha ranged from 0.35 to 0.78 (mean = 0.5) at Brancaster Overy Staithe, from 0.28 to 1.32 (mean = 0.55) at Burnham Overy Staithe and from 0.28 to 0.74 (mean = 0.47) at Thornham (Fig. 7c).

### Foraminiferal Abundance and Environmental Variables

The observed abundance of the three main foraminiferal species was analysed by constructing a generalized additive model (GAM) for each species. The model was constructed to test for significant hypotheses accounting for site difference, date of collection and each of the environmental variables (seasonal trends of each of the environmental variables are shown in Fig. S2). Initially, all environmental variables were included in the model and the selection was based on examining foraminiferal abundance versus environmental variables through multiple regressions. The chosen GAM was the one which produced the lowest AIC (Akaike information criterion) values and the most significant *p* values.

### *Haynesina germanica*

**Fig. 8:**
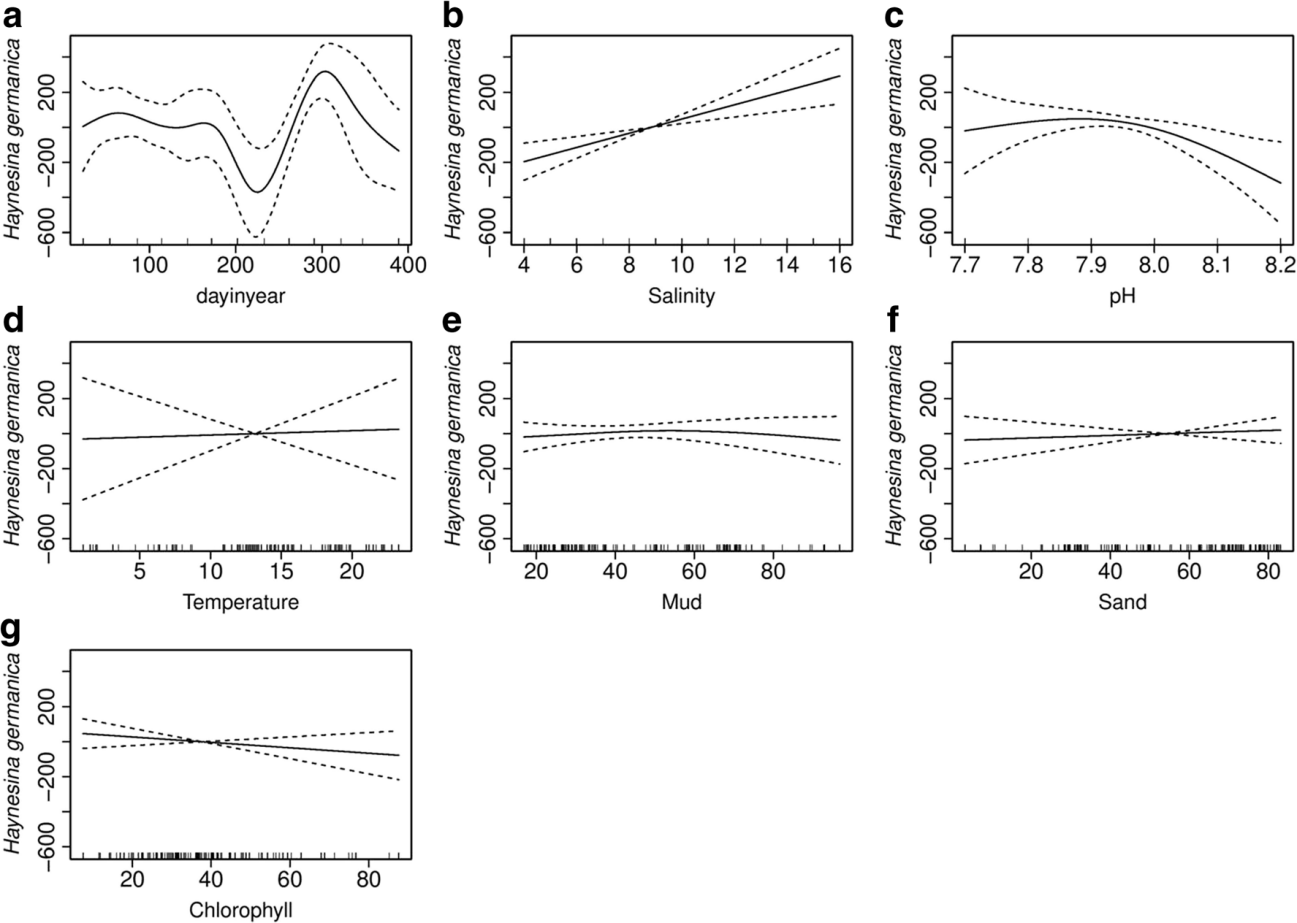
GAM analysis showing smoothed curve of the additive effect to the estimated abundance of *Haynesina germanica* for the individual environmental variables. *Dotted lines* represent 95 % confidence intervals; *marks along the lower axis* represent a single observation. **a** Sampling date. **b** Salinity. **c** pH. **d** Temperature. **e** Mud. **f** Sand. **g** Chlorophyll

*Haynesina germanica* has a mean abundance of 631, 556 and 557 (standard deviation of 43) individuals per 22 cm^3^ at Brancaster Overy Staithe, Burnham Overy Staithe and Thornham, making up 79.7, 85.9 and 93 % of the total number of individuals, respectively. The total number of *H. germanica* was compared between sites, time of collection as well as its interactive response with each of the environmental variables in multiple regression GAM analysis. The GAM regression analysis has shown pH and salinity as the significant variables. The total variation in the *H. germanica* abundance explained by the final chosen model was 55.6 %, and most of this variation in abundance was attributed to the time of collection, with 35.2 % of the total explained deviance in this species abundance (Fig. 8).

### *Ammonia* sp.

**Fig. 9:**
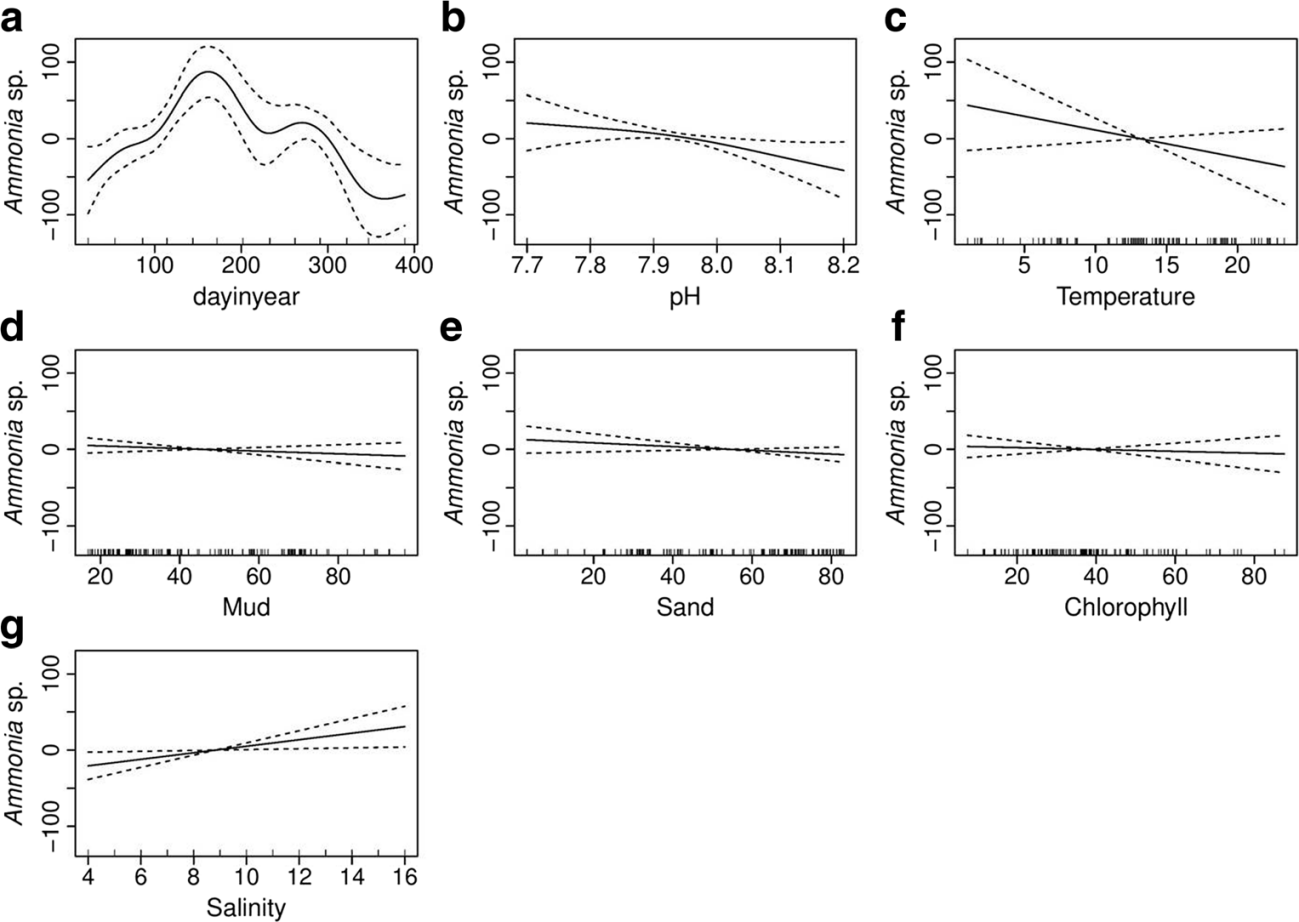
GAM analysis showing smoothed curve of the additive effect to the estimated abundance of *Ammonia* sp. for the individual environmental variables. *Dotted lines* represent 95 % confidence interval; *marks along the lower axis* represent a single observation. **a** Sampling date. **b** pH. **c** Temperature. **d** Mud. **e** Sand. **f** Chlorophyll. **g** Salinity

*Ammonia* sp. has an average relative abundance of 139, 56 and 38 (standard deviation of 53) individuals per 22 cm^3^ sediment samples and comprising approximately 17.5, 8.7 and 6.3 % of the total number of individuals at Brancaster Overy Staithe, Burnham Overy Staithe and Thornham, respectively. Multiple regression GAM analysis showed that site, salinity and pH variables are the significant environmental variables that have an effect on *Ammonia* sp. abundance. The remaining environmental variable predictors were not significant when all included in a single GAM (Fig. 9). The total explained variation of the chosen GAM was 69.3 %, and amongst sites, difference contribution to the model fit was 31.4 %.

### *Elphidium williamsoni*

**Fig. 10:**
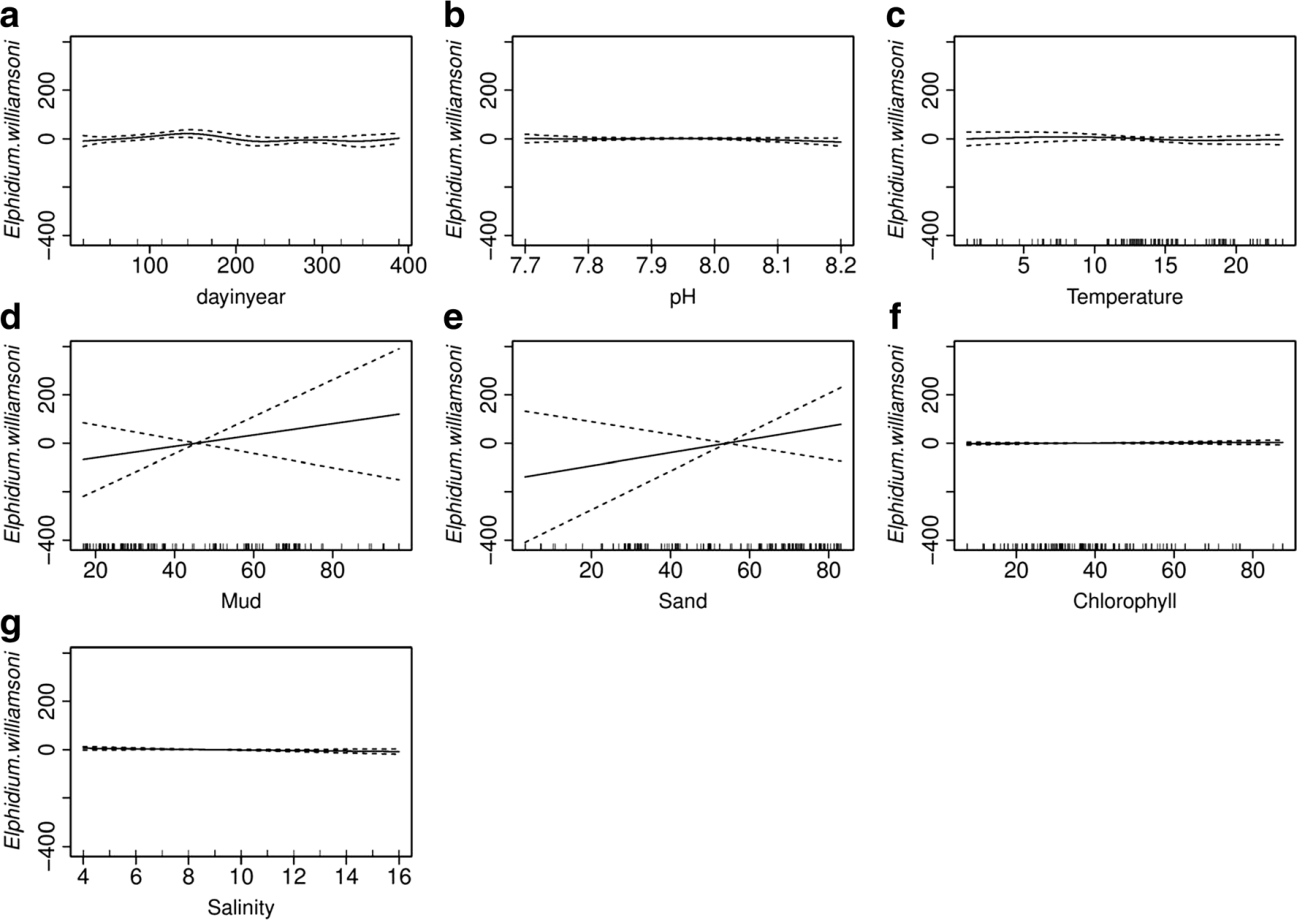
GAM analysis showing smoothed curve of the additive effect to the estimated abundance of *Elphidium williamsoni* for the individual environmental variables. *Dotted lines* represent 95 % confidence interval; *marks along the lower axis* represent a single observation. **a** Sampling date. **b** pH. **c** Temperature. **d** Mud. **e** Sand. **f** Chlorophyll. **g** Salinity

The three sites—Brancaster Overy Staithe, Burnham Overy Staithe and Thornham—have *E. williamsoni* abundance at an average of 20, 32 and 2 (standard deviation of 15) individuals per 22 cm^3^ of sediment, which constitute 2.5, 5 and 0.33 % of the total number of individuals, respectively. Multiple regression GAM analysis showed that sediment grain size is a significant variable (Fig. 10). Amongst sites, differences account for 15.1 % of the 47 % of the total variation explained by the model, suggesting the lack of homogeneity between sites in terms of this species abundance. By far, the largest explained variation of 23.5 % was attributed to the time of sample collection (month of the year) followed by the sediment type variable.

## Discussion

### Patchiness of Foraminiferal Assemblages

To account for the small-scale patchiness in foraminifera distribution, the use of replicate samples was an essential step in obtaining reliable information and confirming that foraminiferal abundance has not been biased by small-scale patchiness. Unlike other studies of foraminifera where patchiness in spatial distribution was observed on a very small scale [2, 18, 24, 36–38], we found that the difference in species frequencies amongst replicate samples in the three areas under study, Brancaster Overy Staithe, Burnham Overy Staithe and Thornham, was not significant. This is evident from the high percentage of affinity reported for both Brancaster Overy Staithe and Thornham and, to a lesser extent, for Burnham Overy Staithe replicates. The affinity indices between replicate samples were 89–97 % for Brancaster Overy Staithe, 89–99 % for Thornham and 83–97 % for Burnham Overy Staithe. These findings differ from other studies. Buzas and Severin [39] found that samples collected from two stations about 10 m apart on Indian River, Florida, contained different foraminifera assemblages. Similarly, Hohenegger et al. [37] reported patchy distributions on a 3 × 3-m scale in the majority of commonly occurring foraminiferal species in the Gulf of Trieste, Adriatic Sea. Murray [40] also considered that the observed significant differences in the monthly records of foraminiferal species from the Exe Estuary could possibly be caused by the patchiness in the distribution patterns, with this clumped distribution pattern of foraminifera being mainly attributed to the spatial distribution of food resources, such as algae, as well as competition between foraminiferal species. Our data have shown that chlorophyll is unevenly distributed across two of the sampled localities, Burnham Overy Staithe and Thornham (correlation values between the three replicate samples ranged between 0.008 and 0.438), yet this has no significant effect on the foraminiferal abundance in our study. This perhaps suggests that spatial difference in food resource distribution is not the responsible factor for the clumped distribution of foraminifera normally observed in the intertidal zones.

### Comparison Between Sites

The foraminiferal composition of the three studied sites comprises *Ammonia* sp., *H. germanica* and *E. williamsoni*, as well as a few individuals of *Quinqueloculina* sp. and *T. inflata*. This foraminiferal association has been found broadly around the coastline of Great Britain, e.g. Dovey Estuary [10], Norfolk [41], Chichester Harbour [18], Erme River, Devon [19], Plym Estuary [20] and Tees Estuary [4, 21], though the exact fauna composition may vary. Despite the absence of patchiness of the foraminiferal assemblages amongst replicate samples in the examined sites of the present study, a profound difference in the faunal composition between Brancaster Overy Staithe, Burnham Overy Staithe and Thornham can be identified. To account for site difference hypotheses in the faunal composition, the GAM was run on the three main species. For both *Ammonia* sp. and *E. williamsoni*, there were significant differences in their observed abundance amongst the three examined sites. There was a generally greater relative abundance of *Ammonia* sp. at Brancaster Overy Staithe compared to both Burnham Overy Staithe and Thornham. *Ammonia* sp. seasonal trend was also slightly different at Burnham Overy Staithe, with its minor spring peak occurring as early as March as opposed to May for both Brancaster Overy Staithe and Thornham. Conversely, the relative abundance of *E. williamsoni* was higher in Burnham Overy Staithe compared to the other two sites. Seasonal patterns in the relative abundance of *E. williamsoni* also tend to vary, with spring and autumn peaks being observed only in Brancaster Overy Staithe and Burnham Overy Staithe. At Thornham, the relative abundance of *E. williamsoni* was consistent throughout the examined period and did not seem to vary with the seasonal cycle.

### Seasonal Trend of Abundance

The average foraminiferal abundance of the low marsh zones of the North Norfolk coast showed similar seasonal variation patterns in foraminifera abundance. The abundance booms occurred at the same period at the three sites, indicating that the desirable conditions for reproduction are related to a more general environmental condition as opposed to conditions specific to each site. The three sites displayed a main peak in autumn (September–October) and another minor peak in late spring (May). The lowest abundance occurred in late summer, between July and August. In agreement with the current study findings, Murray and Alve and Swallow [6, 18] similarly reported high foraminiferal abundances in autumn and spring on the intertidal zone of the Hamble Estuary at Warsash, Hampshire, and Mill Rythe Creek on Chichester Harbour, England, with a summer decline in abundance in June–July and August, respectively. The foraminifera therefore seem to be reproducing rapidly in the spring and autumn months, with the general increase in abundance during the spring and autumn indicative of a stressed environmental condition during the summer and winter months that support only a limited number of individuals. However, contrary to these observations, the average abundance of foraminifera in the low marsh areas of the Cowpen Marsh, Tees Estuary, England, reached its maximum between May and August, whereas the reduction period occurred in November to March [4].

*H. germanica* dominated the three low marsh sites, Brancaster Overy Staithe, Burnham Overy Staithe and Thornham, commonly comprising more than 85.6 % of the living assemblage. Morvan et al. and Castignetti [20, 24] also reported that *H. germanica* was the dominant species forming 89 and 87 % of the total foraminiferal assemblage in the Plym Estuary, South West England, and the Bay of Bourgneuf, France, respectively. In our study, the abundance of *H. germanica* peaked from September to November (autumn), just after a major reduction in abundance in summer (July–August). In contrast, the highest values in the abundance of adult *H. germanica* individuals were reported throughout the spring in the Hamble Estuary, Mill Rythe Creek on Chichester Harbour and the Plym Estuary, England [6, 18, 20]. The summer decline in *H. germanica* abundance observed in North Norfolk was consistent with a similar decline in both the Hamble Estuary and the Plym Estuary, England [20, 22].

*Ammonia* sp. followed basically the seasonal trend pattern for *H. germanica*, but with generally higher abundance in the spring than in the autumn. *Ammonia* sp. abundance over the whole investigation period was very low during the winter at all sites, but it increased generally in the late winter and early spring. The relative abundance then reached its maximum in May. Thereafter, there was a major drop in abundance in the late summer, around July to August, before another minor peak observed in autumn, between September and October. Likewise, Murray and Alve and Swallow [6, 18] found that *Ammonia beccarii* dominated the intertidal stations on the Hamble Estuary, Hampshire, and Mill Rythe Creek on Chichester Harbour, England, from January to May, with the largest relative abundance values occurring between May and June. In the Erme Estuary, South West England, *A. beccarii* was the least abundant species, and it is present only in the spring at OW15 station and in the autumn at OW14 [19]. A population of *A. beccarii* on the Plym Estuary, England, however, displayed low abundance during May and June, but it increased dramatically in October, congruent with this study [20].

*E. williamsoni* was constantly of lower abundance than the other two species, *H. germanica* and *Ammonia* sp. A parallel pattern in seasonal changes was seen in both *Ammonia* sp. and *E. williamsoni*. The abundance exhibited two peaks, one in spring and the other in autumn, at Brancaster Overy Staithe and Burnham Overy Staithe. The significant reduction in abundance occurred in late summer. Horton and Murray [4] noted that on the low marsh zone of Cowpen Marsh, Tees Estuary, England, *E. williamsoni* was most abundant during May and June.

The two minor species, *Quinqueloculina* sp. and *T. inflata*, did not show evidence of any seasonal pattern throughout the investigated period at all three sites. This is due to the relatively low number of encountered individuals from each species at each site. In Guadiana Estuary (Southwestern Iberian Peninsula), both *T. inflata* and *Jadammina macrescens* were rare species in the samples collected at the river mouth, with low total abundance of 3 %, suggesting some level of test export from the nearby habitats might have happened through the tidal currents and flood events [7]. Strong tidal currents can sweep through the intertidal marshes and carry over live foraminifera between distinct foraminiferal associations [7].

### Species Abundance and Environmental Variable Relationship

The various environmental variables that have been previously assessed to have potential impacts on the abundance and composition of the benthic foraminifera assemblage in the intertidal zones are hydrodynamic conditions, vegetation cover, salinity, temperature, organic content, sediment and availability of oxygen [3, 6–8]. In this study, the abundance of the three main foraminiferal species studied in the three sites on the North Norfolk coast are highly correlated, implying a single response to the same abiotic or biotic factors. Both *Ammonia* sp. and *E. williamsoni* abundance peaked in the spring and autumn, whereas *H. germanica* maximum abundance occurred mainly in autumn.

The generally higher assemblage abundance of species could be attributed to the food availability in the environment. The fact of combined blooms in both the chlorophyll values and foraminifera species abundance in the spring and autumn at Brancaster Overy Staithe, in the spring at Burnham Overy Staithe and in the autumn at Thornham indicates that the food supplements might be amongst the important factors that control foraminiferal species abundance on the North Norfolk coast. *Ammonia tepida* abundance from the Ubatuba Bay, Brazil, showed a positive correlation with the chlorophyll concentrations, as shown by the Pearson correlation of 0.60 [42]. On the Long Island Sound, USA, the relative abundance of *Eggerella advena* has decreased in response to changes in the phytoplankton community and the composition of food supply [43]. Burone [42] observed an increase in chlorophyll at the beginning of spring, but it suffered reduction at the end of summer and during autumn. Here, the GAM hypothesis to test for chlorophyll effect as one of the limiting environmental factors on the three main species abundance was not significant at all sites. Murray and Alve [6] also noticed that at neither of the two stations on the Hamble estuary, Hampshire, England, was there any correlation between foraminifera abundance and the chlorophyll content of the sediment.

The total foraminiferal assemblage and abundance of individual species in the North Norfolk intertidal zone, however, seems to be determined by the sediment characteristics. For example, the total number of foraminiferal individuals in one of the Burnham Overy Staithe replicates, A, was the lowest amongst the three replicates throughout the investigated period. Sediment size analysis showed that replicate A contains 75 % sand, whereas the other two replicates, B and C, have averages of 57 and 70 % sand, respectively. Thomas et al. [43] have also reported the absence of foraminifera species in coarse sandy sediment from the eastern Long Island Sound, USA. Here, GAM analysis testing has predicted that sand percentage in the sediment was the only important factor in explaining a portion of the observed variation in the abundance of *E. williamsoni. E. williamsoni* was also seen to be less dominant at Thornham compared to both Brancaster Overy Staithe and Burnham Overy Staithe. Sediment grain size analysis showed that Thornham sediment samples contain a high proportion of mud (68 %) as opposed to 34 and 31 % at Brancaster Overy Staithe and Burnham Overy Staithe, respectively. Likewise, Alve and Murray [22] found that *E. williamsoni* was less dominant in the intertidal zone of the Hamble Estuary, England, because of the generally high mud content of the sediment (63–74 %).

Salinity has been described as a key factor in controlling the faunal composition of the saltmarsh [44, 45]. The recorded salinity range showed subtle changes during the investigation period. It ranged from 4 to –11 ppt (mean = 7 ppt) at Brancaster, from 4 to 12 ppt (mean = 7 ppt) at Burnham and from 8 to 16 ppt (mean = 11 ppt) at Thornham. It is clear that Thornham has the highest salinity mean amongst the three sites throughout the year. Low salinity usually resulted either from runoff from the adjacent land in the form of river discharges, as in the case of Burn River at Burnham Overy Staithe, or sometimes from springs, as might be expected at Brancaster Overy Staithe. The GAM analysis has predicted the significance of salinity in explaining some of the seasonal observed variations in the abundance of *H. germanica* and *Ammonia* sp., but its role was absent in *E. williamsoni*. In Cowpen Marsh (Tees Estuary), only the two dominant foraminiferal species, *H. germanica* and *E. williamsoni*, on the lower marsh showed a clear relationship with salinity [4]. Lastly, although there is non-significance of the temporal changes in the pH values throughout the year, the GAM analysis considers pH as one of the significant factors in explaining some of the seasonal changes in the abundance of both *Ammonia* sp. and *H. germanica*. This is not surprising given the correlation between pore water pH and salinity.

The study showed that air temperature has followed the expected seasonal cycle, where it was 19 °C in late spring (May), 22 °C in late summer (August) and 1 °C in the winter. It has been suggested that an increase in temperature leads to an increase in nutrient concentration and feeding resources needed by foraminiferal species (though nutrient uptake in summer will lead ultimately to a decline in nutrient concentration) [42]. The second potential effect of temperature is its control on the reproduction rate in foraminifera. Even though foraminiferal juvenile individuals are present throughout the year, their percentage has been shown to increase in months of higher temperature [1]. For example, an increase in *A. beccarii* abundance has been reported when temperature was within the range 20–25 °C [46]. Considering that, the major reduction in the abundance of the three main species of this study, however, was observed in late summer, between July and August, when the temperature was in its optimal range (22–23 °C) at all three sites. The GAM analysis further confirmed the non-significance of temperature in explaining the variability of abundance of the three main species at the three sites. One of the reasons could be the excess of organic matter that may lead to extreme oxygen depletion in the sediment and unfavourable eutrophication, making the environment uninhabitable for most foraminiferal fauna [42, 47].

Salinity, pH and sediment size were all significant in our GAM analysis, but still explain only some of the observed variations in species abundance. The source of the remaining unexplained variation in species seasonal abundance is not known. Basson and Murray [23] stated no obvious environmental cause for the rapid increase in the abundance and species diversity in the intertidal environment in Bahrain. Likewise, Alve and Murray and Duijnstee et al. [22, 48] noticed nearly no correlation between the abundance of the most common species and the measured environmental parameters in the northern Adriatic Sea and Hamble Estuary, Hampshire, England, respectively. Buzas [36] has also pointed to the non-significant contribution of the examined environmental variables in explaining the observed variability in foraminifera abundance in the Indian River Lagoon, Florida. The various environmental variables have been thought of as not independent factors and the influence of any particular variable is linked to others [49]. Therefore, the biological response of foraminifera to environmental changes seems to be complex and hardly understood.

## Conclusions

In this work, a time series study over a period of 1 year of the intertidal zone of North Norfolk, UK, was accomplished to investigate the temporal and spatial variability of living foraminiferal assemblages in light of the recorded changes in the environmental variables. Three low marsh sites—Brancaster Ovary Staithe, Burnham Ovary Staithe and Thornham—were studied. Benthic foraminiferal fauna seasonal changes were described in terms of the temporal variability of abundance, spatial variation, patchy distribution, species diversity and the potential effect of different environmental conditions. The total foraminiferal assemblage abundance as well as the abundance for individual species revealed similar seasonal trends across sites. The largest living foraminiferal assemblage abundance was observed in autumn, between September and October, with another peak in late spring (May). The lowest values in the average abundance, however, occurred in late summer, between July and August. These results are largely in agreement with previous studies on the seasonal variability of foraminifera at other sites on the coastline of Great Britain. There are three main species dominating the foraminiferal communities on the North Norfolk coastline. These are *Ammonia* sp., *Haynesina germanica* and *Elphidium williamsoni*, as well as a few individuals of *Quinqueloculina* sp. and *Trochammina inflata. H. germanica* comprises more than 85.6 % of the living assemblage, followed by *Ammonia* sp. (11.4 %) and *E. williamsoni* (2.8 %). Despite the overall similarity in seasonal trends, both *Ammonia* sp. and *E. williamsoni* abundances were the highest throughout spring, as opposed to autumn for *H. germanica*. These differences in the response time of the three main species suggest that each of them might have its preferred reproduction period. Because of the apparent low or absence of patchiness in foraminiferal species distribution amongst the replicate samples, it was possible to identify differences in the foraminiferal assemblage composition amongst the three studied sites when a generalized additive model (GAM) was constructed to test for site difference hypotheses. The difference was evident in the case of *Ammonia* sp. and *E. williamsoni* relative abundance. *Ammonia* sp. relative abundance appeared to be higher at Brancaster Overy Staithe than at both Burnham Overy Staithe and Thornham. *E. williamsoni* was found to be present more in Burnham Overy Staithe samples. The significant difference between sites was also observed when considering changes in the seasonal trends of both *Ammonia* sp. and *E. williamsoni*. For example, *Ammonia* sp. seasonal trend showed its minor spring peak as early as March at Burnham Overy Staithe, as opposed to May for both Brancaster Overy Staithe and Thornham. A hypothetical testing of the significance of each environmental variable measured in this study using GAM analysis has predicted that salinity, pH and sediment grain size are the most influential ecological factors in explaining some of the observed changes in the seasonal trends of the three main species. Both salinity and pH are significant in the case of *H. germanica* and *Ammonia* sp., whilst sediment grain size was significant in explaining some of the seasonal variations in *E. williamsoni*. The remaining environmental factors were not significant. It is only by including more environmental factors that the relative importance of the different ecological controls on the seasonal trends of foraminiferal species in intertidal zones can be properly determined.

## Electronic Supplementary Material

Below is the link to the electronic supplementary material.

ESM 1 (PDF 353 kb)

ESM 2 (PDF 64.1 kb)

ESM 3 (PDF 210 kb)
