# Supplementary material for: Seasonal and Spatial Variations of Saltmarsh Benthic Foraminiferal Communities from North Norfolk, England
Source: Microb Ecol. 2016 Nov 26;73(3):539–55. doi: 10.1007/s00248-016-0895-5 (PMC5348568; doi:10.1007/s00248-016-0895-5)
Supplement: Supplementary file 1 — (PDF 353 kb) [file 248_2016_895_MOESM1_ESM.pdf]

Seasonal and spatial variation of saltmarsh benthic foraminiferal communities from North Norfolk, England

Microbial Ecology Journal

Salha A. Saad and Christopher M. Wade

sale12302006@yahoo.com; Chris.Wade@nottingham.ac.uk

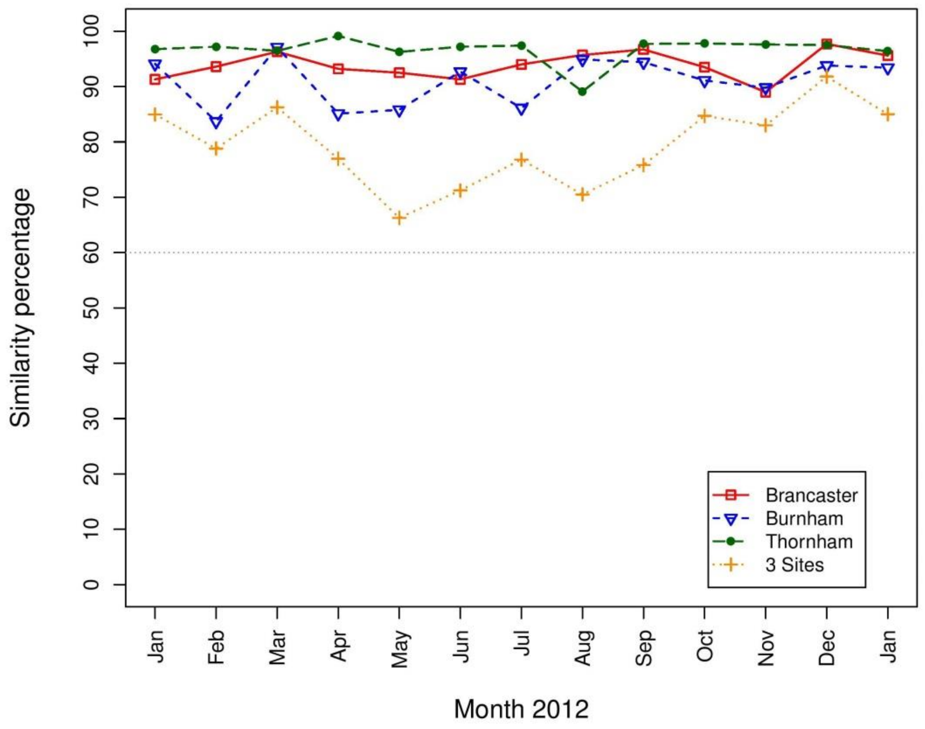

Figure. S1 Similarity of species frequencies between replicate samples of each site and between the three sites, Brancaster, Burnham and Thornham, measured by the Index of Affinity.

Table S1 ANOVA analysis on the replicate samples with each site.

Replicate	Df	Sum of	Mean	F	p-value
Brancaster	2	109074	54537	2.439	0.0922
Burnham	2	498262	249131	9.274	0.000197
Thornham	2	5456	2728	0.485	0.617

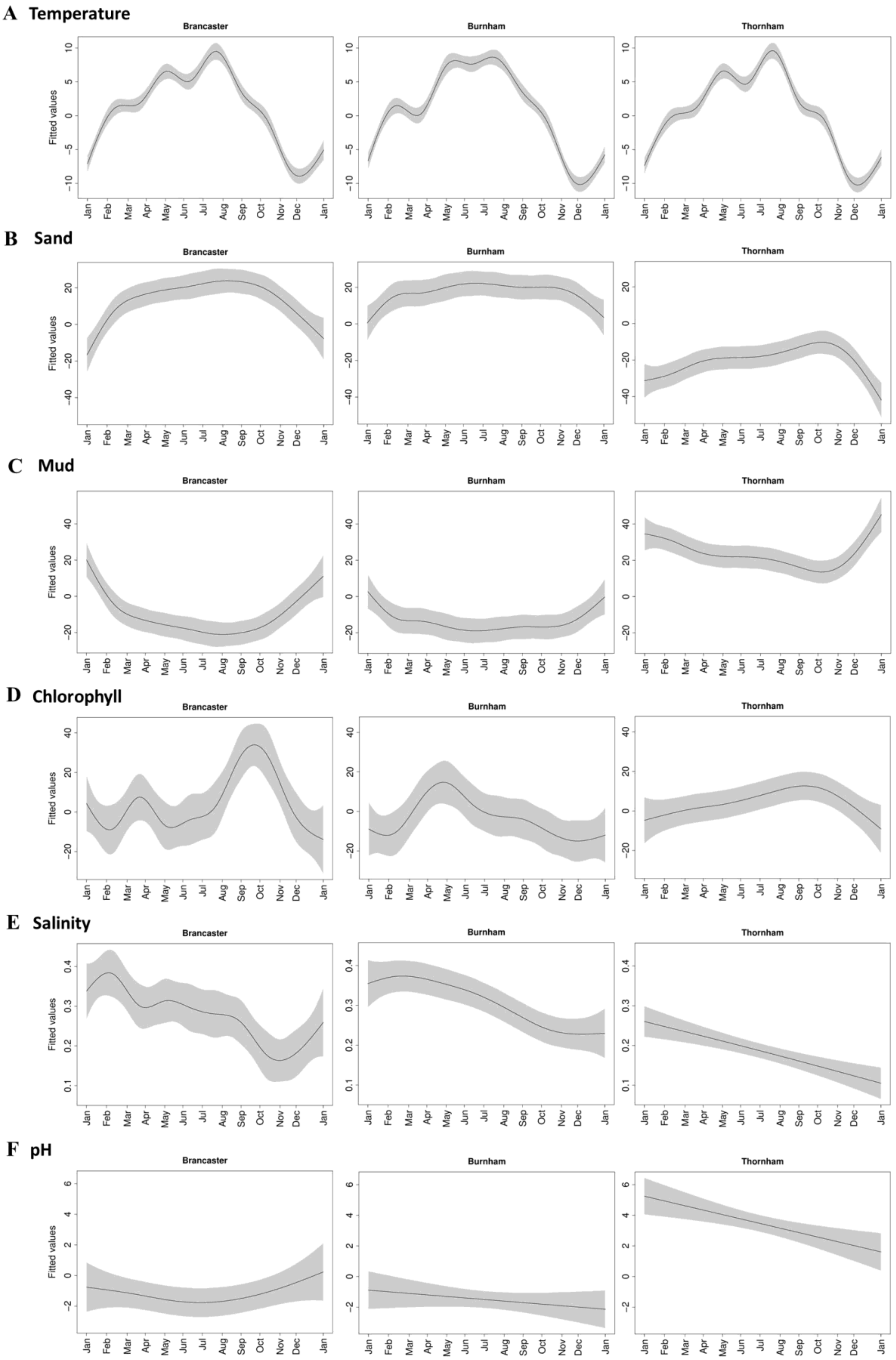
A Temperature
Brancaster
Burnham
Thornham
Fitted values
10
5
0
−5
−10
B Sand
Brancaster
Burnham
Thornham
Fitted values
20
0
−20
−40
C Mud
Brancaster
Burnham
Thornham
Fitted values
40
20
0
−20
D Chlorophyll
Brancaster
Burnham
Thornham
Fitted values
40
20
0
−20
E Salinity
Brancaster
Burnham
Thornham
Fitted values
0.4
0.3
0.2
0.1
F pH
Brancaster
Burnham
Thornham
Fitted values
6
4
2
0
−2
Jan
Feb
Mar
Apr
May
Jun
Jul
Aug
Sep
Oct
Nov
Dec
Jan

Figure S2 The line is the predicated value of each environmental variable from the model and the grey area represents 95% confidence interval. **A: temperature** (The mud temperature showed a seasonal variation as might be expected with the season. The maximum temperature was recorded in August (Summer) at all three sites; 22°C at Brancaster Overy Staithe, 21°C at Burnham Overy Staithe and 22°C at Thornham. The minimum temperature was observed in December; 3.2°C at Brancaster Overy Staithe, 1.7°C at Burnham Overy Staithe and 1.5°C at Thornham), **B: sand** (the average percentage of sand (particles >63 µm) varied little over the year. The sediment samples from Brancaster Overy Staithe and Burnham Overy Staithe were uniform and have very similar percentage of sand with 65% and 68% respectively. Thornham had only 31% sand), **C: mud** (The average percentage of silt- and clay-size particles in the examined sediment (particles <63 µm) varied little over the year at each site. Mud constitutes about 34% of the sediment at Brancaster Overy Staithe, 31.7% at Burnham Overy Staithe and 68.1% at Thornham indicating that the three sites are different in terms of sediment type. Thornham can be classified as sandy mud sediment whereas Brancaster Overy Staithe and Burnham Overy Staithe can be classified as muddy sand sediment.), **D: chlorophyll** (The average active chlorophyll annual trend tends to differ among the three sites. They range from 21 to 75mg/m^2 at Brancaster Overy Staithe, from 12 to 66mg/m^2 at Burnham Overy Staithe and from 15 to 70mg/m^2 at Thornham. Both Brancaster Overy Staithe and Burnham Overy Staithe showed a Spring bloom between March and April with a value of 48 mg/m^2 and 51 mg/m^2 respectively. At Brancaster Overy Staithe, there was a second bloom in Autumn between September and October with an average value of 75.6 mg/m^2. A similar increase of 61 mg/m^2 was observed in October at Thornham), **E: salinity** (At Brancaster Overy Staithe and Burnham Overy Staithe, there was an average salinity of 7‰ compared to 12‰ in Thornham. These values did seem to vary considerably from one month to another. However, Thornham showed a clear seasonal trend in the salinity values that tend to decline gradually throughout the year with the highest values recorded in January (12‰) and the lowest recorded in December (8 ‰), **F: pH** (The sediment pH values have a range of 7.9-8.1 at Brancaster Overy Staithe, 7.8-8.2 at Burnham Overy Staithe and 7.8-8 at Thornham. Seasonally, the pH values varied only slightly among sites, declining gradually from January to December when the lowest value of 7.8 was recorded)
