# Supplementary material for: Seasonal and Spatial Variations of Saltmarsh Benthic Foraminiferal Communities from North Norfolk, England
Source: Microb Ecol. 2016 Nov 26;73(3):539–55. doi: 10.1007/s00248-016-0895-5 (PMC5348568; doi:10.1007/s00248-016-0895-5)
Supplement: Supplementary file 2 — (PDF 64.1 kb) [file 248_2016_895_MOESM2_ESM.pdf]

Table S2: Number of living individuals of *Ammonia* sp., *Haynesina germanica*, *Elphidium williamsoni*, *Quinqueloculina* sp. and *Trochammina inflata* sampled from an area of 22 cm^3 for a one year period from the North Norfolk coastline along with their relative percentage of abundance.

Date	Site	Replicate	Total	No species	*Ammonia* sp.		*Haynesina germanica*		*Elphidium williamsoni*		*Quinqueloculina* spp.		*Trochammina inflata*	
					No	%	No	%	No	%	No	%	No	%
23/01/2012	Brancaster	A	778	4	62	7.9	710	91	5	0.6	1	0.12	0	0
23/01/2012	Brancaster	B	800	4	64	8	717	89.9	17	2	2	0.25	0	0
23/01/2012	Brancaster	C	537	3	85	15.8	445	82.8	7	1.3	0	0	0	0
23/02/2012	Brancaster	A	465	4	54	11.6	402	86.4	8	1.7	1	0.21	0	0
23/02/2012	Brancaster	B	444	3	78	17.5	361	81	5	1	0	0	0	0
23/02/2012	Brancaster	C	671	3	96	14	560	83.4	15	2.2	0	0	0	0
26/03/2012	Brancaster	A	679	3	94	13.8	568	83.6	17	2.5	0	0	0	0
26/03/2012	Brancaster	B	833	3	99	11.8	714	85.7	20	2.4	0	0	0	0
26/03/2012	Brancaster	C	649	4	68	10.4	565	87	15	2.3	1	0.15	0	0
23/04/2012	Brancaster	A	947	4	196	20.6	714	75	36	3.8	1	0.1	0	0
23/04/2012	Brancaster	B	440	3	80	18	348	79	12	2.7	0	0	0	0
23/04/2012	Brancaster	C	981	3	153	15.5	778	79	50	5	0	0	0	0
23/05/2012	Brancaster	A	329	3	99	30	211	64	19	5.7	0	0	0	0
23/05/2012	Brancaster	B	815	4	281	34.4	489	60	44	5	1	0.12	0	0
23/05/2012	Brancaster	C	866	3	239	27.5	549	63	78	9	0	0	0	0
20/06/2012	Brancaster	A	563	4	159	28	361	64	42	7.4	1	0.17	0	0
20/06/2012	Brancaster	B	1042	3	234	22.4	754	72	54	5	0	0	0	0
20/06/2012	Brancaster	C	741	4	224	30	475	64	37	4.9	0	0	5	0.67
20/07/2012	Brancaster	A	597	5	152	25.4	424	71	14	2.3	3	0.5	4	0.67
20/07/2012	Brancaster	B	1851	4	498	26.9	1330	71.8	22	1	0	0	1	0.054
20/07/2012	Brancaster	C	467	5	139	29.7	317	67.8	4	0.8	5	1	2	0.42
20/08/2012	Brancaster	A	376	4	117	31	255	67.8	3	0.7	0	0	1	0.26
20/08/2012	Brancaster	B	212	4	64	30	141	66.5	6	2.8	0	0	1	0.47
20/08/2012	Brancaster	C	259	4	82	31.6	168	64.8	8	3	0	0	1	0.38
18/09/2012	Brancaster	A	590	3	167	28	418	70.8	5	0.8	0	0	0	0
18/09/2012	Brancaster	B	237	3	62	26	172	72.5	3	1.2	0	0	0	0

Continued on Next Page. . .

Table S2 Continued

Date	Site	Replicate	Total	No species	*Ammonia* sp.		*Haynesina germanica*		*Elphidium williamsoni*		*Quinqueloculina* spp.		*Trochammina inflata*	
					No	%	No	%	No	%	No	%	No	%
18/09/2012	Brancaster	C	775	4	215	27.7	542	69.9	16	2	0	0	2	0.25
17/10/2012	Brancaster	A	1247	5	202	16	1013	81	22	1.7	2	0.16	8	0.64
17/10/2012	Brancaster	B	884	3	138	15.6	725	82	21	2.3	0	0	0	0
17/10/2012	Brancaster	C	1287	5	143	11	1121	87	20	1.5	1	0.07	2	0.15
17/11/2012	Brancaster	A	1271	3	109	8	1149	90.4	13	1	0	0	0	0
17/11/2012	Brancaster	B	2165	4	396	18	1734	80	34	1.5	0	0	1	0.046
17/11/2012	Brancaster	C	745	4	91	12	629	84.4	24	3.2	0	0	1	0.13
12/12/2012	Brancaster	A	1052	3	84	7.9	946	89.9	22	2	0	0	0	0
12/12/2012	Brancaster	B	640	3	57	8.9	577	90	6	0.9	0	0	0	0
12/12/2012	Brancaster	C	390	4	34	8.7	347	88.9	8	2	1	0.25	0	0
29/01/2013	Brancaster	A	646	3	47	7	587	90.8	12	1.8	0	0	0	0
29/01/2013	Brancaster	B	2070	3	215	10	1832	88.9	23	1.1	0	0	0	0
29/01/2013	Brancaster	C	531	3	46	8.6	465	87.5	20	3.7	0	0	0	0
23/01/2012	Burnham	A	56	5	3	5	49	87.5	2	3.5	1	1.78	1	1.78
23/01/2012	Burnham	B	479	3	31	6.4	440	91.8	8	1.6	0	0	0	0
23/01/2012	Burnham	C	960	3	70	7	870	90.6	20	2	0	0	0	0
23/02/2012	Burnham	A	103	4	6	5.8	79	76.6	17	16.5	0	0	1	0.97
23/02/2012	Burnham	B	526	4	37	7	481	91.4	7	1.3	1	0.19	0	0
23/02/2012	Burnham	C	1239	4	92	7.4	1119	90	27	2	1	0.08	0	0
26/03/2012	Burnham	A	270	5	12	4.4	230	85	24	8.8	1	0.37	3	1.11
26/03/2012	Burnham	B	1758	4	116	6.5	1558	88.6	82	4.6	2	0.11	0	0
26/03/2012	Burnham	C	1398	4	127	9	1202	85.9	66	4.7	3	0.21	0	0
23/04/2012	Burnham	A	161	3	12	7.4	141	87.5	8	4.9	0	0	0	0
23/04/2012	Burnham	B	486	3	79	16	354	72.8	53	10.9	0	0	0	0
23/04/2012	Burnham	C	1000	4	149	14.9	787	78.7	62	6.2	2	0.2	0	0
23/05/2012	Burnham	A	458	4	28	6	346	75.5	83	18	1	0.21	0	0
23/05/2012	Burnham	B	954	4	135	14	747	78	71	7.4	0	0	1	0.1
23/05/2012	Burnham	C	1856	4	340	18	1345	72.4	168	9	3	0.16	0	0
20/06/2012	Burnham	A	477	4	34	7	404	84.6	38	7.9	0	0	1	0.2
20/06/2012	Burnham	B	287	4	38	13	227	79	21	7.3	0	0	1	0.34
20/06/2012	Burnham	C	671	4	88	13	537	80	44	6.5	0	0	2	0.29
20/07/2012	Burnham	A	247	4	23	9	217	87.8	6	2.4	0	0	1	0.4
20/07/2012	Burnham	B	265	3	49	18.4	198	74.7	18	6.7	0	0	0	0
20/07/2012	Burnham	C	273	3	51	18.6	213	78	9	3.2	0	0	0	0

Table S2 Continued

Date	Site	Replicate	Total	*No species*	*Ammonia sp.*		*Haynesina germanica*		*Elphidium williamsoni*		*Quinqueloculina* spp.		*Trochammina inflata*	
					No	%	No	%	No	%	No	%	No	%
20/08/2012	Burnham	A	67	3	6	8.9	58	86.5	3	4.4	0	0	0	0
20/08/2012	Burnham	B	245	3	31	12.6	204	83	10	4	0	0	0	0
20/08/2012	Burnham	C	226	3	25	11	194	85.8	7	3	0	0	0	0
18/09/2012	Burnham	A	194	5	14	7	171	88	6	3	1	0.51	2	1.03
18/09/2012	Burnham	B	708	3	79	11	616	87	13	1.8	0	0	0	0
18/09/2012	Burnham	C	656	3	64	9.7	562	85.6	30	4.5	0	0	0	0
17/10/2012	Burnham	A	695	3	53	7.6	586	84	56	8	0	0	0	0
17/10/2012	Burnham	B	1142	3	88	7.7	1025	89.7	29	2.5	0	0	0	0
17/10/2012	Burnham	C	1109	4	52	4.6	1017	91.7	36	3.2	0	0	4	0.36
17/11/2012	Burnham	A	703	4	44	6	596	84.7	62	8.8	0	0	1	0.14
17/11/2012	Burnham	B	839	3	35	4	778	92.7	26	3	0	0	0	0
17/11/2012	Burnham	C	878	3	33	3.7	832	94.7	13	1.4	0	0	0	0
12/12/2012	Burnham	A	215	4	16	7.4	193	89.7	4	1.8	0	0	2	0.93
12/12/2012	Burnham	B	720	3	39	5.4	672	93	9	1.2	0	0	0	0
12/12/2012	Burnham	C	882	3	26	2.9	834	94.5	22	2.4	0	0	0	0
29/01/2013	Burnham	A	438	4	19	4	373	85	45	10.2	1	0.22	0	0
29/01/2013	Burnham	B	700	4	37	5	631	90	31	4.4	0	0	1	0.14
29/01/2013	Burnham	C	893	3	39	4	813	91	41	4.5	0	0	0	0
23/01/2012	Thornham	A	433	3	21	4.8	410	94.6	2	0.46	0	0	0	0
23/01/2012	Thornham	B	605	3	15	2	589	97	1	0.16	0	0	0	0
23/01/2012	Thornham	C	648	3	18	2.7	627	96.7	3	0.46	0	0	0	0
23/02/2012	Thornham	A	596	4	17	2.8	575	96.7	3	0.5	1	0.16	0	0
23/02/2012	Thornham	B	456	3	21	4.6	434	95	1	0.21	0	0	0	0
23/02/2012	Thornham	C	972	4	20	2	949	97.6	2	0.2	1	0.1	0	0
26/03/2012	Thornham	A	981	4	25	2.5	947	96.5	7	0.7	2	0.2	0	0
26/03/2012	Thornham	B	646	3	18	2.7	627	97	1	0.15	0	0	0	0
26/03/2012	Thornham	C	655	4	33	5	615	93.8	2	0.3	5	0.76	0	0
23/04/2012	Thornham	A	665	3	32	4.8	632	95	1	0.15	0	0	0	0
23/04/2012	Thornham	B	772	4	34	4.4	734	95	3	0.38	1	0.12	0	0
23/04/2012	Thornham	C	543	3	23	4	519	95.5	1	0.18	0	0	0	0
23/05/2012	Thornham	A	754	5	67	8.8	674	89	2	0.26	10	1.32	1	0.13
23/05/2012	Thornham	B	570	3	42	7	526	92	2	0.35	0	0	0	0
23/05/2012	Thornham	C	780	3	67	8.5	704	90	9	1.15	0	0	0	0
20/06/2012	Thornham	A	1072	4	157	14.6	905	84.4	9	0.83	1	0.093	0	0

Table S2 Continued

Date	Site	Replicate	Total	No species	*Ammonia sp.*		*Haynesina germanica*		*Elphidium williamsoni*		*Quinqueloculina* spp.		*Trochammina inflata*	
					No	%	No	%	No	%	No	%	No	%
20/06/2012	Thornham	B	1017	4	139	13.6	873	85.8	4	0.39	1	0.098	0	0
20/06/2012	Thornham	C	1284	4	161	12.5	1119	87	3	0.23	1	0.077	0	0
20/07/2012	Thornham	A	264	4	33	12.5	230	87	0	0	1	0.37	0	0
20/07/2012	Thornham	B	220	3	33	15	187	85	0	0	0	0	0	0
20/07/2012	Thornham	C	273	4	40	14.6	232	84.9	0	0	1	0.36	0	0
20/08/2012	Thornham	A	305	4	22	7	280	91.8	0	0	0	0	3	0.98
20/08/2012	Thornham	B	163	4	24	14.7	136	83.4	2	1.22	0	0	1	0.61
20/08/2012	Thornham	C	296	3	17	5.7	279	94	0	0	0	0	0	0
18/09/2012	Thornham	A	887	5	54	6	825	93	5	0.56	1	0.11	2	0.22
18/09/2012	Thornham	B	961	5	54	5.6	902	93.8	3	0.31	1	0.1	1	0.1
18/09/2012	Thornham	C	271	3	21	7.7	249	91.8	1	0.36	0	0	0	0
17/10/2012	Thornham	A	502	4	55	10.9	446	88.8	0	0	0	0	1	0.19
17/10/2012	Thornham	B	577	4	28	4.8	542	93.9	1	0.17	0	0	6	1
17/10/2012	Thornham	C	855	5	32	3.7	817	95.5	0	0	1	0.11	5	0.58
17/11/2012	Thornham	A	745	4	37	4.9	705	94.6	2	0.26	0	0	1	0.13
17/11/2012	Thornham	B	911	3	35	3.8	871	95.6	5	0.54	0	0	0	0
17/11/2012	Thornham	C	910	5	28	3	875	96	0	0	1	0.1	6	0.65
12/12/2012	Thornham	A	580	3	24	4	554	95.5	2	0.34	0	0	0	0
12/12/2012	Thornham	B	235	4	7	2.9	227	96.5	0	0	0	0	1	0.42
12/12/2012	Thornham	C	339	4	17	5	321	94.6	0	0	1	0.29	0	0
29/01/2013	Thornham	A	190	3	6	3	184	96.8	0	0	0	0	0	0
29/01/2013	Thornham	B	168	3	6	3.5	162	96.4	0	0	0	0	0	0
29/01/2013	Thornham	C	250	3	0	0	249	99.6	1	0.4	0	0	0	0
