# Supplementary material for: Seasonal and Spatial Variations of Saltmarsh Benthic Foraminiferal Communities from North Norfolk, England
Source: Microb Ecol. 2016 Nov 26;73(3):539–55. doi: 10.1007/s00248-016-0895-5 (PMC5348568; doi:10.1007/s00248-016-0895-5)
Supplement: Supplementary file 3 — (PDF 210 kb) [file 248_2016_895_MOESM3_ESM.pdf]

Table S3: Measurements of the six environmental variables taken for one year period from the North Norfolk coastline.

Date	Site	Replicate	Temperature (°C)	Sand (%)	Mud (%)	Sediment Texture	Chlorophyll a (mg/m^3)	pH	Salinity (‰)
23/01/2012	Brancaster	A	5.6	52.4	47.7	Muddy Sand	38.8	8	8
23/01/2012	Brancaster	B	5.6	30.9	69.1	Sandy Mud	40.3	8	7
23/01/2012	Brancaster	C	5.6	7.1	93	Mud	47.6	8	7
23/02/2012	Brancaster	A	12	51.1	48.9	Muddy Sand	14.2	8.2	8
23/02/2012	Brancaster	B	11.9	73	27	Muddy Sand	19.7	8.1	8
23/02/2012	Brancaster	C	12.5	66.6	33.4	Muddy Sand	38.2	8.1	7
26/03/2012	Brancaster	A	14	72.7	27.3	Muddy Sand	33.6	8	6
26/03/2012	Brancaster	B	14.2	62.4	37.6	Muddy Sand	40.3	7.9	10
26/03/2012	Brancaster	C	15.7	57.8	42.2	Muddy Sand	40	8	7
23/04/2012	Brancaster	A	14.8	73.2	26.7	Muddy Sand	33	8	10
23/04/2012	Brancaster	B	15.1	68.1	32	Muddy Sand	48.8	8	10
23/04/2012	Brancaster	C	15.5	60.4	39.5	Muddy Sand	58.2	8	9
23/05/2012	Brancaster	A	19	71.6	28.3	Muddy Sand	19.7	8	5
23/05/2012	Brancaster	B	18.8	72.4	27.6	Muddy Sand	17.9	8	6
23/05/2012	Brancaster	C	19.6	71.8	28.2	Muddy Sand	40.6	8	5
20/06/2012	Brancaster	A	18.4	72.1	28	Muddy Sand	22.7	8	4
20/06/2012	Brancaster	B	18.1	75.2	24.8	Muddy Sand	28.2	8	7
20/06/2012	Brancaster	C	18.5	68.9	31	Muddy Sand	38.2	8	7
20/07/2012	Brancaster	A	18.9	73.1	26.9	Muddy Sand	33	7.9	5
20/07/2012	Brancaster	B	19.1	70.2	29.8	Muddy Sand	44.9	7.9	10
20/07/2012	Brancaster	C	19	70.2	24.2	Muddy Sand	47.9	8	7
20/08/2012	Brancaster	A	22.1	79.7	20.2	Muddy Sand	39.4	7.9	7
20/08/2012	Brancaster	B	22.2	79.1	21.1	Muddy Sand	41.8	8	7
20/08/2012	Brancaster	C	21.7	79.1	20.9	Muddy Sand	34.8	8	6
18/09/2012	Brancaster	A	15.4	79.1	20.9	Muddy Sand	85.2	7.9	8
18/09/2012	Brancaster	B	15.8	69.8	30.2	Muddy Sand	54.3	8.1	4
18/09/2012	Brancaster	C	16.4	69.9	30.1	Muddy Sand	68.8	7.9	10
17/10/2012	Brancaster	A	12.3	76.7	23.3	Muddy Sand	71.3	7.8	6

Continued on Next Page. . .

Table S3 Continued

Date	Site	Replicate	Temperature (°C)	Sand (%)	Mud (%)	Sediment Texture	Chlorophyll a (mg/m^3)	pH	Salinity (‰)
17/10/2012	Brancaster	B	13.4	71.7	28.3	Muddy Sand	74.9	7.8	9
17/10/2012	Brancaster	C	13	78.1	22	Muddy Sand	59.4	8	6
17/11/2012	Brancaster	A	8.6	55.5	44.5	Muddy Sand	52.7	7.8	9
17/11/2012	Brancaster	B	8.7	73.9	26.2	Muddy Sand	38.2	8	5
17/11/2012	Brancaster	C	8.7	79.8	20.2	Muddy Sand	55.8	7.9	6
12/12/2012	Brancaster	A	3.1	49.5	50.5	Sandy Mud	23.9	7.8	11
12/12/2012	Brancaster	B	3.1	62.7	37.3	Muddy Sand	31.2	8	5
12/12/2012	Brancaster	C	3.5	59.4	40.5	Muddy Sand	36.7	7.8	7
29/01/2013	Brancaster	A	7.5	49.8	50.2	Sandy Mud	27.3	8	10
29/01/2013	Brancaster	B	8.4	72.5	27.5	Muddy Sand	25.1	7.9	7
29/01/2013	Brancaster	C	8	39.1	60.9	Sandy Mud	20	7.9	10
23/01/2012	Burnham	A	6	65.8	34.1	Muddy Sand	27.6	8	6
23/01/2012	Burnham	B	6.3	59.8	40.1	Muddy Sand	11.5	8	7
23/01/2012	Burnham	C	6	22.8	77.2	Sandy Mud	52.7	8	12
23/02/2012	Burnham	A	13	83.1	16.9	Muddy Sand	21.5	8.2	4
23/02/2012	Burnham	B	13.1	76.9	23.1	Muddy Sand	20.3	8.1	8
23/02/2012	Burnham	C	11.9	49.8	50.2	Sandy Mud	16	8	11
26/03/2012	Burnham	A	14.6	82.6	17.4	Muddy Sand	38.5	8.2	5
26/03/2012	Burnham	B	14	58.3	41.7	Muddy Sand	25.1	8	11
26/03/2012	Burnham	C	14.2	65	35	Muddy Sand	20	8	10
23/04/2012	Burnham	A	14.6	65.4	34.6	Muddy Sand	53	8.1	5
23/04/2012	Burnham	B	12.6	68.9	31.1	Muddy Sand	67.9	8	10
23/04/2012	Burnham	C	13.2	50.2	49.8	Muddy Sand	33.3	8	8
23/05/2012	Burnham	A	19.8	81.4	18.6	Muddy Sand	75.8	8	5
23/05/2012	Burnham	B	19.9	75.5	24.4	Muddy Sand	33.3	8	6
23/05/2012	Burnham	C	20.1	69.1	30.8	Muddy Sand	31.2	7.9	7
20/06/2012	Burnham	A	19.9	82.2	17.8	Muddy Sand	44.6	8.1	5
20/06/2012	Burnham	B	22.3	70.8	29.1	Muddy Sand	31.5	8	6
20/06/2012	Burnham	C	22.2	66.6	33.5	Muddy Sand	57.3	8	8
20/07/2012	Burnham	A	19.1	82	18	Muddy Sand	32.4	8.1	5
20/07/2012	Burnham	B	19.8	77.9	22.1	Muddy Sand	30	8	5
20/07/2012	Burnham	C	19.6	68.6	31.4	Muddy Sand	22.7	8	10
20/08/2012	Burnham	A	21	78.7	21.3	Muddy Sand	29.7	8	5
20/08/2012	Burnham	B	21.7	75.3	24.7	Muddy Sand	52.1	8	6
20/08/2012	Burnham	C	21.2	62.8	37.2	Muddy Sand	29.4	7.9	11

Table S3 Continued

Date	Site	Replicate	Temperature (°C)	Sand (%)	Mud (%)	Sediment Texture	Chlorophyll a (mg/m^3)	pH	Salinity (‰)
18/09/2012	Burnham	A	15.5	77.6	22.5	Muddy Sand	25.4	8	5
18/09/2012	Burnham	B	15.9	70.9	29.1	Muddy Sand	44.9	8	9
18/09/2012	Burnham	C	15.9	66.7	33.2	Muddy Sand	36.1	8	8
17/10/2012	Burnham	A	12.1	75.6	24.5	Muddy Sand	24.2	8	5
17/10/2012	Burnham	B	12.7	70.2	29.7	Muddy Sand	16.9	7.8	7
17/10/2012	Burnham	C	12.8	68.6	31.4	Muddy Sand	26.3	7.8	10
17/11/2012	Burnham	A	7.6	80.5	19.4	Muddy Sand	34.8	8	4
17/11/2012	Burnham	B	7.5	75.2	24.8	Muddy Sand	27.6	8	5
17/11/2012	Burnham	C	7.6	64.5	35.5	Muddy Sand	26	7.8	5
12/12/2012	Burnham	A	1.9	73.7	26.4	Muddy Sand	11.8	8	7
12/12/2012	Burnham	B	1.7	71.1	28.9	Muddy Sand	19.1	7.9	6
12/12/2012	Burnham	C	1.7	63.1	36.9	Muddy Sand	16	7.8	5
29/01/2013	Burnham	A	6.9	55.1	44.9	Muddy Sand	19.7	8	7
29/01/2013	Burnham	B	6.9	68.4	31.6	Muddy Sand	21.5	7.9	6
29/01/2013	Burnham	C	7.3	40.4	59.6	Sandy Mud	36.4	7.9	11
23/01/2012	Thornham	A	5.6	31.7	68.3	Sandy Mud	24.5	7.9	12
23/01/2012	Thornham	B	5.6	10.3	89.8	Sandy Mud	34.2	8	12
23/01/2012	Thornham	C	4.7	26.9	73.1	Sandy Mud	31.8	7.9	12
23/02/2012	Thornham	A	11	13.5	86.5	Sandy Mud	37.3	7.9	16
23/02/2012	Thornham	B	11.5	28.3	71.7	Sandy Mud	40.3	7.9	15
23/02/2012	Thornham	C	10.9	17.7	82.3	Sandy Mud	28.8	7.9	12
26/03/2012	Thornham	A	12.9	22.7	77.2	Sandy Mud	53	7.9	14
26/03/2012	Thornham	B	13.3	22.4	77.6	Sandy Mud	7.5	7.9	11
26/03/2012	Thornham	C	13.6	37.7	62.3	Sandy Mud	46.1	8	16
23/04/2012	Thornham	A	15.9	42	58	Sandy Mud	62.8	7.9	12
23/04/2012	Thornham	B	14.6	34.3	65.7	Sandy Mud	37	7.9	15
23/04/2012	Thornham	C	15.2	31.5	68.5	Sandy Mud	38.2	8	11
23/05/2012	Thornham	A	19.3	31.1	68.9	Sandy Mud	29.7	7.9	14
23/05/2012	Thornham	B	17.1	25.5	74.5	Sandy Mud	37.6	7.9	10
23/05/2012	Thornham	C	19.2	42.4	57.6	Sandy Mud	29.1	7.9	15
20/06/2012	Thornham	A	18.8	28.6	71.3	Sandy Mud	33.6	7.9	15
20/06/2012	Thornham	B	19.1	30.9	69.1	Sandy Mud	22.4	7.9	14
20/06/2012	Thornham	C	19.2	41.4	58.6	Sandy Mud	56.4	7.9	14
20/07/2012	Thornham	A	18.4	39.6	60.5	Sandy Mud	48.2	7.9	10
20/07/2012	Thornham	B	17.9	29.2	70.8	Sandy Mud	42.4	7.8	11

Table S3 Continued

Date	Site	Replicate	Temperature (°C)	Sand (%)	Mud (%)	Sediment Texture	Chlorophyll a (mg/m^3)	pH	Salinity (‰)
20/07/2012	Thornham	C	18.3	29.8	70.2	Sandy Mud	62.8	7.9	11
20/08/2012	Thornham	A	23.3	44.1	55.9	Sandy Mud	25.4	7.8	13
20/08/2012	Thornham	B	21.5	33.6	66.4	Sandy Mud	42.7	7.9	8
20/08/2012	Thornham	C	22.8	32.3	67.7	Sandy Mud	33.6	7.9	10
18/09/2012	Thornham	A	14.5	41.2	58.8	Sandy Mud	34.2	7.9	11
18/09/2012	Thornham	B	14.3	31.9	68.1	Sandy Mud	87.6	7.8	15
18/09/2012	Thornham	C	14	34	66	Sandy Mud	41.5	7.9	9
17/10/2012	Thornham	A	12.3	48.3	51.6	Sandy Mud	60.3	7.9	9
17/10/2012	Thornham	B	11.9	47.7	52.3	Sandy Mud	47.6	7.8	13
17/10/2012	Thornham	C	12.1	38.5	61.5	Sandy Mud	76.7	7.8	13
17/11/2012	Thornham	A	8.6	32.6	67.4	Sandy Mud	47.6	7.8	12
17/11/2012	Thornham	B	8.7	41.8	58.2	Sandy Mud	52.7	7.8	13
17/11/2012	Thornham	C	8.6	49.9	50.2	Sandy Mud	14.5	7.9	10
12/12/2012	Thornham	A	1	46.7	53.3	Sandy Mud	37.6	7.8	8
12/12/2012	Thornham	B	2	29.7	70.3	Sandy Mud	30.3	7.8	8
12/12/2012	Thornham	C	1.5	29.5	70.6	Sandy Mud	49.7	7.8	11
29/01/2013	Thornham	A	6.3	10.8	89.3	Sandy Mud	30.9	7.8	10
29/01/2013	Thornham	B	7.4	7.2	92.8	Mud	17.9	7.8	8
29/01/2013	Thornham	C	6.4	3.2	96.8	Mud	22.4	7.7	13
